# Supplementary material for: An alpha-helical lid guides the target DNA toward catalysis in CRISPR-Cas12a
Source: Nat Commun. 2024 Feb 17;15:1473. doi: 10.1038/s41467-024-45762-6 (PMC10874386; doi:10.1038/s41467-024-45762-6)
Supplement: Supplementary file 1 — Supplementary Information [file 41467_2024_45762_MOESM1_ESM.pdf]

Supplementary Information

An Alpha-helical Lid Guides the Target DNA toward Catalysis in CRISPR-Cas12a

Aakash Saha, Mohd Ahsan,† Pablo R. Arantes,† Michael Schmitz, Christelle Chanez, Martin Jinek and Giulia Palermo*

Table of Contents

Supplementary Methods......2
Structural models......2
Molecular Dynamics (MD) simulations......4
Principal Component Analysis......5
Umbrella Sampling simulations......6
Calculation of bending angles......8
Alchemical free energy simulations......8
FnCas12a expression and purification......9
FnCas12a nuclease activity assays......10
Supplementary Figures......11
Supplementary Tables......32
Supplementary References......35

Corresponding author:
Dr. Giulia Palermo (giulia.palermo@ucr.edu)
†These authors contributed equally.

Supplementary Methods

Structural models. Molecular simulations were based on two structures of the *F. novicida* Cas12a obtained upon NTS cleavage: (1) the cryo-EM structure EMD-0065 (PDB: 6GTG [https://doi.org/10.2210/pdb6GTG/pdb] (Cas12a – I4 Conformation))[1] solved at 3.27 Å resolution and (2) the X-ray structure PDB: 5NFV [https://doi.org/10.2210/pdb5NFV/pdb] (FnCas12a bound to R-loop),[2] solved at 2.50 Å resolution. The 6GTG [https://doi.org/10.2210/pdb6GTG/pdb] (Cas12a – I4 Conformation) cryo-EM structure contains an α-helical "lid" (residues L1008 – K1021, Fig. 1a), majority of which is structurally disordered in the 5NFV [https://doi.org/10.2210/pdb5NFV/pdb] (FnCas12a bound to R-loop) X-ray structure. Hence, the missing N1009 – F1017 residues in the 5NFV [https://doi.org/10.2210/pdb5NFV/pdb] (FnCas12a bound to R-loop) structure, along with other missing loops, were reconstructed through homology modelling using the SWISS-MODEL software[3,4] (Fig. 1b), where we used the X-ray 5NFV [https://doi.org/10.2210/pdb5NFV/pdb] (FnCas12a bound to R-loop) structure as the template.[4]

We built the complete Cas12a system (Fig. 1c) starting from the 6GTG [https://doi.org/10.2210/pdb6GTG/pdb] (Cas12a – I4 Conformation)[1] cryo-EM structure of Cas12a (Fig. 1a), in which the lid displays an α-helical form. Since 6GTG [https://doi.org/10.2210/pdb6GTG/pdb] (Cas12a – I4 Conformation) lacks the 5'-tail of the TS (bases from -1 to -7) downstream of the crRNA:TS duplex, containing the scissile phosphate,[5] we built it from the 5NFV [https://doi.org/10.2210/pdb5NFV/pdb] (FnCas12a bound to R-loop) X-ray structure of Cas12a (Fig. 1b) that contains a longer TS. In detail, we performed an all-Cα based RMSD fitting of the 6GTG [https://doi.org/10.2210/pdb6GTG/pdb] (Cas12a – I4 Conformation) structure on 5NFV [https://doi.org/10.2210/pdb5NFV/pdb] (FnCas12a bound to R-loop) (RMSD = ~2 Å, Supplementary Fig. 2a). Next, we performed a separate backbone based RMSD fitting (RMSD = ~2.5 Å) of the nucleic acids between the two structures and patched the TS:NTS hybrid downstream of the crRNA:TS duplex from the 5NFV [https://doi.org/10.2210/pdb5NFV/pdb] (FnCas12a bound to R-loop) structure onto 6GTG [https://doi.org/10.2210/pdb6GTG/pdb] (Cas12a – I4 Conformation). We maintained the nucleic acid sequence of the 6GTG [https://doi.org/10.2210/pdb6GTG/pdb] (Cas12a – I4 Conformation) structure. This complex was further refined using the multi-template mode of the program MODELLER.[6] The RuvC domain of Cas12a is highly conserved across nucleases,[7] including the Cas9 enzyme[8] that was first harnessed for gene editing.[9] It holds a two-metal aided architecture, characterized by the highly conserved DEDD (or DDE) motif,[2] in which Mg^{2+} ions are coordinated by carboxylate groups. The X-ray structure of Cas12i2 (PDB: 6LTU [https://doi.org/10.2210/pdb6LTU/pdb] (Cas12i2 ternary complex)[10]), phylogenetically related to Cas12a,[10] captured the catalytic Mg^{2+} ions coordinating

the DED motif in RuvC (Supplementary Fig. 2b). The two catalytic Mg^{2+} ions were located in the RuvC site of Cas12a by superimposing the DDE motif of the two structures (6GTG [https://doi.org/10.2210/pdb6GTG/pdb] (Cas12a – I4 Conformation) and 6LTU [https://doi.org/10.2210/pdb6LTU/pdb] (Cas12i2 ternary complex)). Finally, the complete Cas12a system was subjected to force field-based vacuum minimization to obtain an energetically stable complex, further used for long timescale MD simulations. Importantly, distance analyses of the DDE motif coordinating with the Mg^{2+} ions along ~10 μs-long MD simulation (Supplementary Fig. 2c) underscores the stability of the two-metal ion architecture in the system.

The catalytically competent state of Cas12a for TS cleavage was built taking cues from related crystal structures of Cas12a orthologues, holding the DNA TS within the RuvC active site. The X-ray structure of the CRISPR-Cas12b (PDB: 5U30 [https://doi.org/10.2210/pdb5U30/pdb] (AacC2c1-sgRNA-extended target DNA ternary complex),[11] at 2.9 Å resolution) accommodates the TS within the RuvC catalytic cleft (Supplementary Fig. 8a). The TS bends down from the crRNA:TS duplex toward the active site, forming a U-shaped strand with its 5'-tail interacting with the Nuc domain. Cas12a and Cas12b share a highly conserved RuvC catalytic domain, which is superposable in the 6GTG [https://doi.org/10.2210/pdb6GTG/pdb] (Cas12a – I4 Conformation) and 5U30 [https://doi.org/10.2210/pdb5U30/pdb] (AacC2c1-sgRNA-extended target DNA ternary complex) structures (Supplementary Fig. 8b). A comparison of the two structures shows that the nucleic acids are also superposable and differ for a longer U-shaped TS that locates with the RuvC active site in Cas12b. Building on this similarity, the 5U30 [https://doi.org/10.2210/pdb5U30/pdb] (AacC2c1-sgRNA-extended target DNA ternary complex) structure was used as a template for biased MD simulations, starting from the complete Cas12a system that includes Mg^{2+} ions in the RuvC site. Restraint forces were applied to reduce the RMSD between initial and final conformations of the DNA nucleobases -1 to -5, which include the scissile phosphate, similar to a prior modeled structure of Cas12a.[2] The following potential was applied:

$$V = \begin{cases} \frac{k}{2N}\left(RMS(t) - RMS^*(t)\right)^2, & RMS(t) > RMS^*(t) \\ 0, & RMS(t) \le RMS^*(t) \end{cases} \qquad (1)$$

where *k* is the collective force constant, *N* is the number of targeted atoms, *RMS(t)* is the actual $RMSD$ between the current (at time *t,* initially) and target configurations, and *RMS*(t)* is reduced with a constant velocity from the initial $RMSD$ value between the initial and target configurations toward the desired final $RMSD$ (i.e., zero). The difference in RMSD was gradually reduced in 30 steps of 20 ns each with a force constant *k* of 30 kcal mol^{-1} $Å^{-2}$, collecting an overall ensemble of ~600 ns. This protocol leads to a relaxed trajectory, allowing the protein to adapt and

accommodate the transition. The resultant structure contains the scissile phosphate, between the nucleobases T_{-2} and G_{-3}, which coordinates the catalytic Mg^{2+} ions (Supplementary Fig. 8c,d). This complex was subjected to ~10 μs of MD simulation for further refinement and to ensure long timescale stability (Supplementary Fig. 9). Along the simulations, the active site is stable (Supplementary Fig. 9c) and the DDE motif steadily coordinates Mg^{2+} (Supplementary Fig. 9d).

All the systems were embedded in explicit waters, while counterions were added to neutralize the total charge at physiological conditions, leading to periodic simulation cells of ~138*149*167 $Å^3$ and a total of ~307,000 atoms for each system.

Molecular Dynamics (MD) simulations. Conventional MD simulations were performed to equilibrate the systems prior to long timescale MD on Anton-2.[12] A simulation protocol tailored for RNA/DNA nucleases was adopted, embracing the use of the Amber ff19SB[13] force field, which includes the ff99bsc1 corrections for DNA[14] and the ff99bsc0+χOL3 corrections for RNA.[15,16] The TIP3P model was employed for explicit water molecules.[17] The Li & Merz 12-6 model of non-bonded interactions was used for Mg^{2+} ions.[18] We have extensively employed these force field models in computational studies of CRISPR-Cas systems,[19] showing also that they perform well for long timescale simulations on Anton-2.[20] The Li & Merz model also reported a good description of Mg^{2+} bound sites, in agreement with quantum/classical simulations.[21] An integration time step of 2 fs was employed. All bond lengths involving hydrogen atoms were constrained using the SHAKE algorithm. Temperature control (300 K) was performed via Langevin dynamics,[22] with a collision frequency γ = 1. Pressure control was accomplished by coupling the system to a Berendsen barostat[23] at a reference pressure of 1 atm and with a relaxation time of 2 ps. The systems were subjected to energy minimization to relax water molecules and counter ions, keeping the protein, the RNA, DNA and Mg^{2+} ions fixed with harmonic position restraints of 300 kcal/mol · $Å^2$. Then, the systems were heated up from 0 to 100 K in a canonical ensemble (NVT), by running two simulations of 5 ps each, imposing position restraints of 100 kcal/mol · $Å^2$ on the above-mentioned elements of the system. The temperature was further increased up to 200 K in ~100 ps of MD in the isothermal-isobaric ensemble (NPT), reducing the restraint to 25 kcal/mol · $Å^2$. Subsequently, all restraints were released, and the temperature of the systems was raised up to 300 K in a single NPT simulation of 500 ps. After ~ 1.1 ns of equilibration, ~10 ns of NPT runs were carried out allowing the density of the systems to stabilize around 1.01 g cm^{-3}. Finally, production runs were carried out in the NVT ensemble, collecting ~120 ns. These simulations have been performed using the GPU-empowered version of AMBER 20.[24] The well-equilibrated systems were used as starting points for simulations on Anton-2.[12]

Long timescale MD simulations of the CRISPR-Cas12a systems were performed using Anton-2,[12] a special-purpose supercomputer for micro-to-millisecond length MD simulations, starting from well-equilibrated configurations, obtained after ~120 ns of conventional MD (*vide supra*). Simulations on Anton-2 were performed using the same force field parameters used for conventional MD simulations. A reversible multiple time step algorithm[25] was employed to integrate the equations of motion with a time step of 2 fs for short-range nonbonded and bonded forces and 6 fs for the long-range nonbonded forces. Simulations were performed in the NPT ensemble using the multigrator integrator as implemented in Anton-2.[26] Pressure control was accomplished via the Martyna, Tobias, Klein (MTK) barostat,[27] set to maintain 1 bar of pressure, with a tau (piston time constant) parameter of 0.0416667 ps and reference temperature of 310.15 K. The barostat period was set to the default value of 480 ps per timestep. Temperature control was accomplished via the Nosé-Hoover thermostat[28,29] with a tau (time constant) parameter of 0.0416667 ps. The k-Gaussian split Ewald method[30] was used for long-range electrostatic interactions. Hydrogen atoms were added assuming standard bond lengths and were constrained to their equilibrium position with the SHAKE algorithm.[31] By using this approach and the Anton-2 supercomputer,[12] each model system was simulated for ~10 μs and in replicates. Specifically, we obtained multiple trajectories of the 6GTG [https://doi.org/10.2210/pdb6GTG/pdb] (Cas12a – I4 Conformation) cryo-EM structure (simulated for ~10 μs) and its more complete model including a longer DNA (~10 μs in two replicates), as well as of the 5NFV [https://doi.org/10.2210/pdb5NFV/pdb] (FnCas12a bound to R-loop) X-ray structure (~10 μs in two replicates). The structural model of the catalytically competent Cas12a including the DNA TS within the RuvC site was also simulated for ~10 μs. This resulted in a total of ~60 μs of all-atom classical MD simulations.

Principal Component Analysis. PCA is a statistical method used to reduce a large number of degrees of freedom to an essential subspace set, which reports on the large-amplitude, collective motions in biological molecules undergoing MD simulations. In PCA, the covariance matrix of the protein Cα atoms is calculated and diagonalized to obtain a new set of coordinates (eigenvectors) to describe the system motions. Each eigenvector – also called Principal Component (PC) – is associated with an eigenvalue corresponding to the mean square fluctuation contained in the system's trajectory projected along that eigenvector. By sorting the eigenvectors according to their eigenvalues, the first PC1 corresponds to the system's largest amplitude motion, and the dynamics of the system along PC1 are usually referred *"essential dynamics".*[32,33]

Here, PCA has been performed considering the simulations of the complete Cas12a after NTS cleavage (Fig. 1c, Supplementary Table 1). First, the collective ensembles were combined

and subjected to RMS-fit to the same reference configuration, removing rotational and translational motions, to ensure a consistent eigenbasis on all compared systems. Then, the principal motions of the protein have been captured starting from the mass-weighted covariance matrix of the Cα atoms. Each element in this matrix is the covariance between atoms i and j, defining the i, j position of the covariance matrix (C_{ij}):

$$C_{ij} = \langle (\vec{r}_i - \langle \vec{r}_i \rangle)(\vec{r}_j - \langle \vec{r}_j \rangle) \rangle \quad (2)$$

where $\vec{r}_i$ and $\vec{r}_j$ are the position vectors of atoms i and j, and the brackets denote an average over the sampled time period. A positive sign of this product indicates that the two atoms move in a correlated manner, otherwise, a negative value points to anti-correlated atoms. If the product is zero, then it evinces that the atoms' displacements are independent of each other. The covariance matrix was then diagonalized, leading to a complete set of orthogonal collective eigenvectors, each associated with a corresponding eigenvalue. The eigenvalues denote how much each eigenvector is representative of the system dynamics, thus giving a measure of the contribution of each eigenvector to the total variance. Indeed, the eigenvectors with the largest eigenvalues correspond to the most relevant motions. By projecting the displacement vectors of each atom along the trajectory onto the eigenvectors (i.e., by taking the dot product between the two vectors at each frame), the PCs were obtained. The first PC1, commonly referred to as *"essential dynamics"*,[32,33] is plotted on the 3D structure of the complete CRISPR-Cas12a system indicating the largest amplitude motions of the protein (Supplementary Fig. 6e). PCA has been performed using cpptraj of AMBER 20,[24] while the Normal Mode Wizard[34] plugin of the Visual Molecular Dynamics[35] program has been used for the graphical rendering.

Umbrella Sampling simulations. The umbrella sampling (US) method[36] was used to compute the free energy profile associated with the traversal of the DNA TS toward the RuvC active site. In this method, several simulations (US windows) are run in parallel with additional harmonic bias potential applied to selected Reaction Coordinates (RCs):

$$V(RC) = \frac{k}{2}(RC(t) - RC^{*})^2 \quad (3)$$

where $V(RC)$ is the value of the bias potential, k is a bias force constant, $RC(t)$ is the value of RC at given time t and RC^* is the reference value of RC. By using different RC^* values in each US window, one can sample the biased probability distribution $p_b(RC)$ along the whole RC range of interest. Thanks to a simple reweighting scheme providing the unbiased probabilities, the free energy of a process along the RCs can be computed (*vide infra*).

We performed two-dimensional Umbrella Sampling (2-D US) simulations at 300 K using two reaction coordinates (RCs). $RC1$ was the difference in root-mean-square deviation ($RMSD$) of all

the Cα atoms between the initial (Fig. 1c) and the final (Supplementary Fig. 8d) states. $RC2$ was the distance between the centre of mass of the RuvC catalytic core (D917, E1006, D1255 coordinating with the two catalytic Mg^{2+} ions) and of the TS region including the scissile phosphate (i.e., the DNA nucleobases at positions -2 to -4, as indicated by biochemical and single-molecule experiments).[5,37] Restraint forces were applied on the two RCs as follows. The difference in $RMSD$ between initial and final conformations was reduced, according to the following potential:

$$V = \{\frac{k}{2N}\left(RMS(t) - RMS^{*}(t)\right)^{2},\ RMS(t) > RMS^{*}(t)\ 0,\ \ RMS(t) \leq RMS^{*}(t) \qquad (4)$$

where *k* is the collective force constant, *N* is the number of targeted atoms, *RMS(t)* is the actual $RMSD$ between the current (at time *t,* initially) and target configurations, and *RMS*(t)* is reduced with a constant velocity from the initial $RMSD$ value (~6 Å) between the initial and target configurations toward the desired final $RMSD$ (i.e., zero). $RC1$ was divided into bin sizes of 0.2 Å $RMSD$ difference, and each window was run for ~10 ns with a collective force constant *k* of 50 kcal mol^{-1} $Å^{-2}$. Additionally, restraint forces were also applied to reduce the $RC2$ from ~30 Å to ~6.4 Å, according to the same equation. $RC2$ was discretized into bin sizes of 0.2 Å and individual windows were run for ~15 ns with a collective force constant *k* of 50 kcal mol^{-1} $Å^{-2}$. Overall, we simulated 28 ($RC1$) * 119 ($RC2$) = 3,332 windows, resulting in a collective ensemble of ~50 μs simulations.

Two independent sets of 2-D US simulations were performed: (i) for the wild-type system and (ii) upon mutating relevant residues of the α-helical lid (N1009, F1012, K1013, R1014, R1016, K1018) into alanine. Then, the free energy profiles were computed using the Weighted Histogram Analysis (WHAM) method[38] upon removing the initial 1/3rd of the US trajectories from each window, corresponding to the relaxation of the system. The unbiased probability distribution ($p_u(RC)$) was recovered from the biased probability distribution ($p_b(RC)$) through the formula:

$$p_u(RC) = p_b(RC) * exp\,(\frac{V(RC)-F}{k_B T}) \qquad (5)$$

where $V(RC)$ is the value of the applied potential at given RC in a given US window, $k_B$is the Boltzmann constant, T is the reference temperature, F corresponds to an expected value of $exp^{V(RC)}$ for each US window and is optimized with the WHAM algorithm.[38] Analysis of the conformational ensembles was performed on the reweighted trajectories using the exponential factor $exp\,(\frac{V(RC)-F}{k_B T})$.

Analysis of the free energy landscape was performed by computing the minimum free energy pathway using an approach similar to the Dijkstra algorithm.[39] In detail, the map is sampled from the origin point, propagating to the lowest energy point available on each iteration until it

reaches the target point. This enabled us to consider both the RCs along a minimum free energy path. The convergence of the free energy profiles was evaluated by plotting the minimum free energy pathways, considering the remaining 10 ns runs from each US window, for ~5 ns through ~10 ns (Supplementary Fig. 12). The convergence is reached ~8 ns onward for both systems and hence we used bin sizes of ~10 ns to compute our 2-D free energy surfaces (Supplementary Fig. 11) and the minimum free energy paths (Fig. 4 and Supplementary Fig. 11). The error estimation on the minimum free energy pathways was performed using the Monte Carlo bootstrap error analysis.[40]

Calculation of bending angles. Conformational changes of the protein and nucleic acids were monitored through four critical bending angles describing: *(i)* crRNA:TS duplex arching, *(ii)* Rec2 bending and *(iii)* Nuc bending, *(iv)* TS bending. In cases *(i)*, *(ii)* and *(iii)*, we computed the angle between two vectors passing through two regions of interest (defined as mask 1 and mask 2, Supplementary Table 2). We generated a plane based on the first frame of MD production runs by fitting the Cα atoms of the residues under selection (or P atoms for the nucleobases) with the smallest mean square error. A vector ($\vec{V}$) was generated through the centre of mass of two critical points of the selection relevant to the conformational change of interest (*viz.,* mask1 to mask2, Supplementary Table 2) on the plane. Another vector ($\overrightarrow{V_{\perp}}$), perpendicular to $\vec{V}$, out of the plane was generated from the frame of reference. The bending angles were studied by calculating the angle of the vector ($\vec{V}$) along the trajectory with respect to $\overrightarrow{V_{\perp}}$. For the calculation of *(iv)* TS bending, we calculated the angle between two vectors ($\overrightarrow{V_1}$ and $\overrightarrow{V_2}$) passing through the centre of mass of two critical points of the selection relevant to the conformational change of interest (*viz.,* mask1 to mask2, Supplementary Table 2).

Alchemical free energy simulations**.** We computed the relative free energy changes (i.e., $\Delta\Delta G$) of the individual residues with respect to alanine in binding the DNA target strand (TS), by performing alchemical free energy simulations.[41] Here, the DNA target strand (TS, ligand) bound to the Cas12a complex (receptor) through the α-helical lid was decoupled to an unbound state following the alchemical free energy formalism using softcore (SC) potentials,[42] by gradually diminishing the electrostatic and van der Waals interactions of the TS (A_{14}, C_2, G_1 and G_{-1}) bases that interact with the lid. We used a lambda (λ) dynamic variable to define the thermodynamic states of the system along this decoupling alchemical pathway. Thermodynamic integration (TI) simulations were performed at 21 λ values from 0 to 1, at an interval of 0.05, for the wild-type (WT) CRISPR-Cas12a and for each individual alanine mutant. The systems were minimized and equilibrated using the simulation protocol described above. The final binding free energies were

computed using the Multistate Bennett Acceptance Ratio (MBAR) method[43,44] to integrate the free energies over the different lambda values.[45,46] The relative binding free energy ($\Delta\Delta G$) was calculated as follows:

$$\Delta\Delta G_{residue} = \Delta G_{WT} - \Delta G_{mutated} \quad (6)$$

In detail, the pairwise interactions of the DNA TS region (A_{14}, C_2, G_1 and G_{-1}) responsible for binding the lid (L1008 to K1021) was "softened" by modifying the Lenard-Jones (LJ) softcore potentials for van der Waal interactions and Coulombic ($Coul$) softcore potentials for electrostatic interactions as follows:

$$U_{LJ}^{SC}(r_{ij};\lambda) = U_{LJ}[r_{ij}^{LJ}(\lambda;\alpha)] \quad (7)$$

and

$$U_{Coul}^{SC}(r_{ij};\lambda) = U_{Coul}[r_{ij}^{Coul}(\lambda;\beta)] \quad (8)$$

where, α and β are adjustable positive semidefinite parameters for the LJ and $Coul$ softcore interactions, respectively, with values of zero corresponding to no softcore modification for λ value. The long-range interaction cut-off, temperature, and pressure were set as described above for Molecular Dynamics simulations. During TI, the potentials were calculated "on the fly", i.e., as the simulation evolved through the different values of the λ parameter that progressively removed the non-bonded interactions of the ligand (i.e., the TS) using softcore potentials to transition from a bound to an unbound state.

For each system (i.e., WT CRISPR-Cas12a, and the N1009A, F1012A, K1013A, R1014A, R1016A, K1018A mutants), we considered two states A and B based on the reaction coordinate values along the minimum free energy path of TS traversal (Fig. 4a). Alchemical simulations were performed at 21 values of λ (from 0 to 1, in steps of 0.05), reaching ~30 ns for each window. Convergence of the $\Delta\Delta G$ for each system is reported in Supplementary Fig. 16. Considering all the systems and states investigated here, a total of ~8.8 μs sampling was reached.

FnCas12a expression and purification. The DNA sequence of *Francisella tularensis* subsp. novicida U112 (Fn)Cas12a (WP_003040289) was codon optimized for heterologous expression in Escherichia coli (*E. coli*) and synthesized by GeneArt (Thermo Fisher Scientific). The FnCas12a gene was inserted into the 1B plasmid (Addgene #29653) using ligation-independent cloning (LIC), resulting in a construct carrying an N-terminal hexahistidine tag followed by a tobacco etch virus (TEV) protease cleavage site. Point mutations were introduced by Gibson assembly of the PCR-amplified vector backbone with gBlock Gene Fragments (IDT) encoding the individual mutations. The sequences of the synthetic gene, primer and gBlocks are listed in Supplementary Data 1. Mutant FnCas12a constructs were purified as for wild type.

Purification of Cas12a was done as described.[47,48] In brief, Cas12a constructs were expressed in *E. coli* BL21 Rosetta2 (DE3) cells (Novagen, Wisconsin, USA). Cells were lysed in 20 mM Tris pH 8.0, 500 mM NaCl, 5 mM imidazole, 1 µg/mL pepstatin, 200 µg/mL 4-(2-Aminoethyl)benzenesulfonyl fluoride hydrochloride (AEBSF) by ultrasonication. Clarified lysate was applied to a 10 ml Ni-NTA (Sigma-Aldrich) affinity column. The column was washed with 20 mM Tris pH 8.0, 500 mM NaCl, 5 mM imidazole, and bound protein was eluted by increasing imidazole concentration to 250 mM. Eluted protein was dialysed against 20 mM HEPES pH 7.5, 250 mM KCl, 1 mM DTT, 1 mM EDTA overnight at 4 °C in the presence of TEV protease to remove the 6xHis- affinity tag. Cleaved protein was further purified using a HiTrap HP Heparin column (GE Healthcare, Illinois, USA), eluting with a linear gradient to 1.0 M KCl. Elution fractions were pooled, concentrated, and further purified by size exclusion chromatography using a Superdex 200 (16/600) column (GE Healthcare) in 20 mM HEPES-KOH pH 7.5, 500 mM KCl, 1 mM DTT yielding pure, monodisperse proteins. Aliquots were flash-frozen in liquid nitrogen and stored at -80°C.

FnCas12a nuclease activity assays . *In vitro* nuclease activity assays were conducted using purified WT or mutant FnCas12a proteins programmed with a crRNA targeting the λ-sequence (oMS017, IDT, HPLC purified) and a dsDNA substrate containing fluorescently labeled TS (oDS285:oDS271, Merck, HPLC purified). FnCas12a and crRNA were mixed in a ratio of 1:1.2 and incubated for 10 min at 25 °C to allow binary complex formation. Reactions were started by addition of target dsDNA (FnCas12a:dsDNA, 10:1) and incubated at 37 °C. All samples were assembled in a final reaction volume of 20 µL containing 0.5 µM (mutant) FnCas12a, 0.6 µM crRNA and 50 nM dsDNA in a final buffer of 10 mM HEPES-KOH pH 7.5, 250 mM KCl, 5 mM $MgCl_2$, 0.5 mM DTT. Reactions were stopped at indicated time points by addition of EDTA and Proteinase K (Thermo Fisher Scientific) in final concentrations of 80 mM and 0.8 mg/mL, respectively and incubated for 15 min at 37 °C. Samples were mixed with equal volume of a 2X dPAGE loading dye (95 % formamide, 25 mM EDTA), heated to 95 °C for 5 min and resolved on a 15 % denaturing (7 M Urea) polyacrylamide gels run in 0.5 X TBE buffer. Assays were conducted in technical triplicates; indicated error bars represent the standard error of mean. Fluorescence of the ATTO532-labeled substrate and cleavage products was detected using a Typhoon FLA 9500 gel imager; the cleavage rate was quantified based on loss of uncleaved substrate DNA using ImageQuant TL v.8.2.0. Sequences of RNA and DNA oligos utilized in the nuclease assays are listed in Supplementary Table 3.

Supplementary Figures

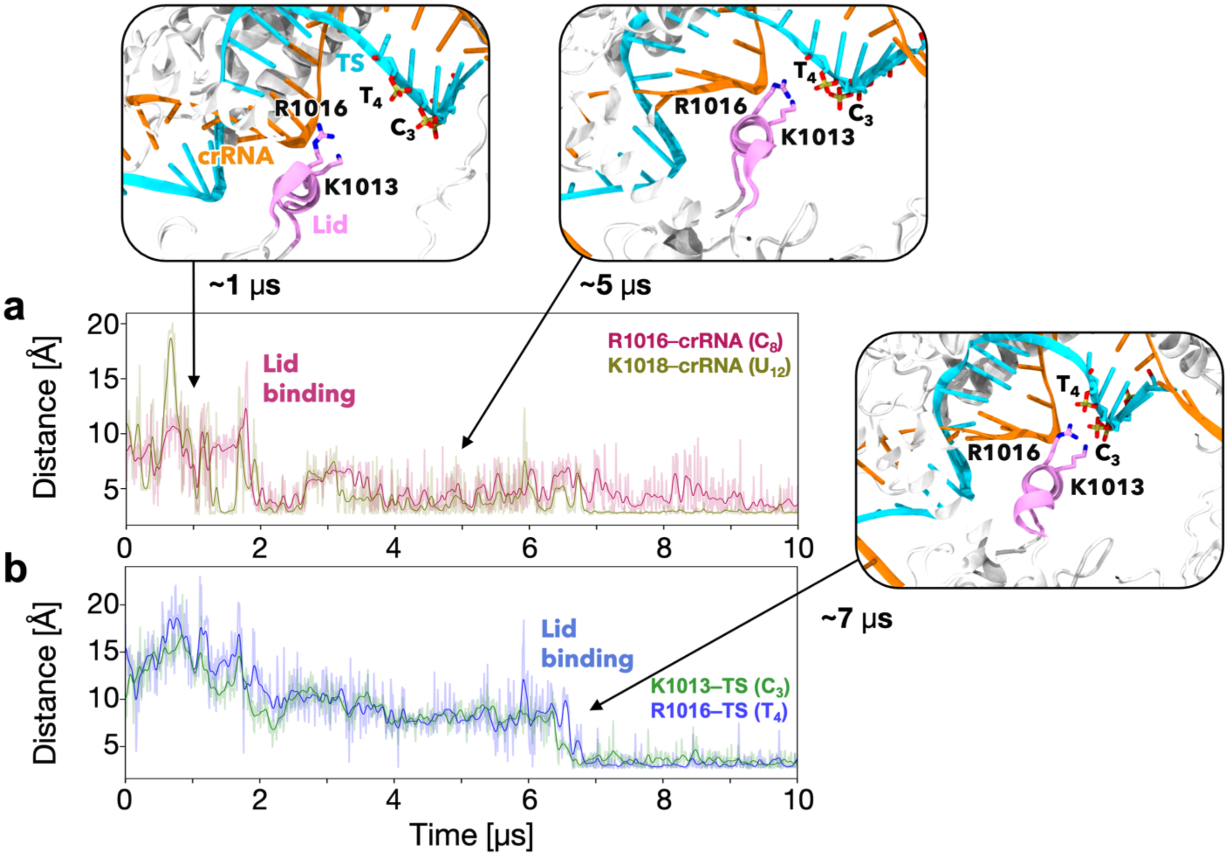

Supplementary Fig. 1: Molecular dynamics (MD) simulations of the 6GTG [https://doi.org/10.2210/pdb6GTG/pdb] (Cas12a – I4 Conformation) **cryo-EM structure of CRISPR-Cas12a (Fig. 1a).** Interactions established by the positively charged residues of the α-helical lid with **(a)** the crRNA (R1016:NH1 – C_8:OP1 and K1018:NZ – U_{12}:OP1) and **(b)** the DNA TS (K1013:NZ – C_3:OP1, R1016:NH2 – T_4:OP1) evolving over ~10 μs of molecular dynamics (MD) simulations. Interactions are measured as oxygen-nitrogen distance and are considered formed under 4 Å distance.[49] Representative snapshots from MD simulations are indicated by the arrows, showing the progressively increasing interaction between the α-helical lid (mauve) and the crRNA:TS duplex (crRNA: orange, TS: cyan; top panel and side panel). The R1016 and K1018 residues form intermittent interactions with the crRNA backbone at positions 8 and 12 respectively, especially after ~4 μs of MD simulations. K1013 and R1016 approach the DNA TS at positions 3 and 4 respectively and form stable electrostatic interactions upon ~7 μs of MD (i.e., persistent interactions through the remainder of the MD run). The translucent lines in the plots show the actual data points, while the solid lines show the running average over 5 ns windows of MD simulation.

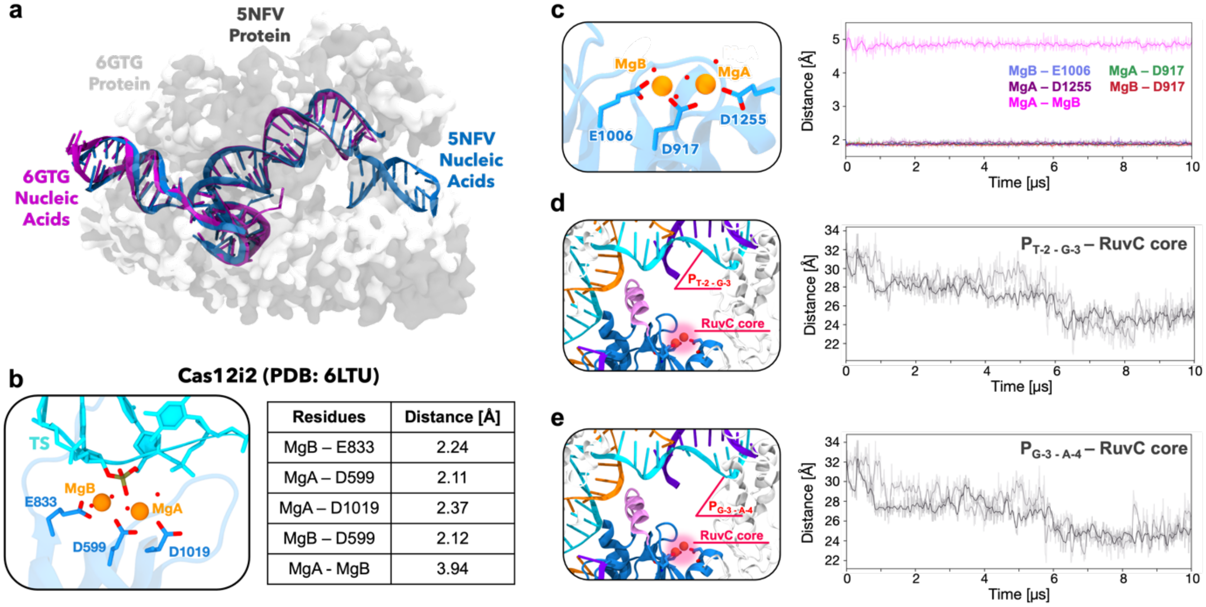

Residues	Distance [Å]
MgB – E833	2.24
MgA – D599	2.11
MgA – D1019	2.37
MgB – D599	2.12
MgA - MgB	3.94

Supplementary Fig. 2: Structure and dynamics of the complete CRISPR-Cas12a system (Fig. 1c). a Superimposition of the cryo-EM structure (PDB: 6GTG [https://doi.org/10.2210/pdb6GTG/pdb] (Cas12a – I4 Conformation)) onto the X-ray structure (PDB: 5NFV [https://doi.org/10.2210/pdb5NFV/pdb] (FnCas12a bound to R-loop)) of CRISPR-Cas12a. The protein is shown in molecular surface (white for 6GTG [https://doi.org/10.2210/pdb6GTG/pdb] (Cas12a – I4 Conformation) and gray for 5NFV [https://doi.org/10.2210/pdb5NFV/pdb] (FnCas12a bound to R-loop)) and the nucleic acids are shown in ribbons (purple for 6GTG [https://doi.org/10.2210/pdb6GTG/pdb] (Cas12a – I4 Conformation) and blue for 5NFV [https://doi.org/10.2210/pdb5NFV/pdb] (FnCas12a bound to R-loop)). More details are in the Supplementary Methods. **b** X-ray structure of CRISPR-Cas12i2 (PDB: 6LTU [https://doi.org/10.2210/pdb6LTU/pdb] (Cas12i2 ternary complex)),[10] capturing the catalytic site of the RuvC domain in the presence of the DNA TS substrate interacting with the catalytic Mg^{2+} ions. Relevant distances of the catalytic architecture are enlisted in the table. **c** Distance analyses between the key catalytic residues (D917, E1006 and D1255) and the two catalytic Mg^{2+} ions (MgA and MgB) evolving over ~10 µs MD simulation, exhibiting noteworthy stability of the catalytic core. A representative snapshot of the RuvC catalytic core with the catalytic Mg^{2+} ions is shown with the graph. **d – e** Distance between the centre of mass of the RuvC catalytic core (left panel, composed of D917, E1006 and D1255 residues coordinating with two Mg^{2+} ions) and the scissile phosphate (PSCI), i.e., **(d)** between T_{-2} and G_{-3}, and **(e)** G_{-3} and A_{-4}, as also shown with representative snapshots. The translucent lines in the plots show the

actual data points, while the solid lines show the running average over 5 ns windows of MD simulation.

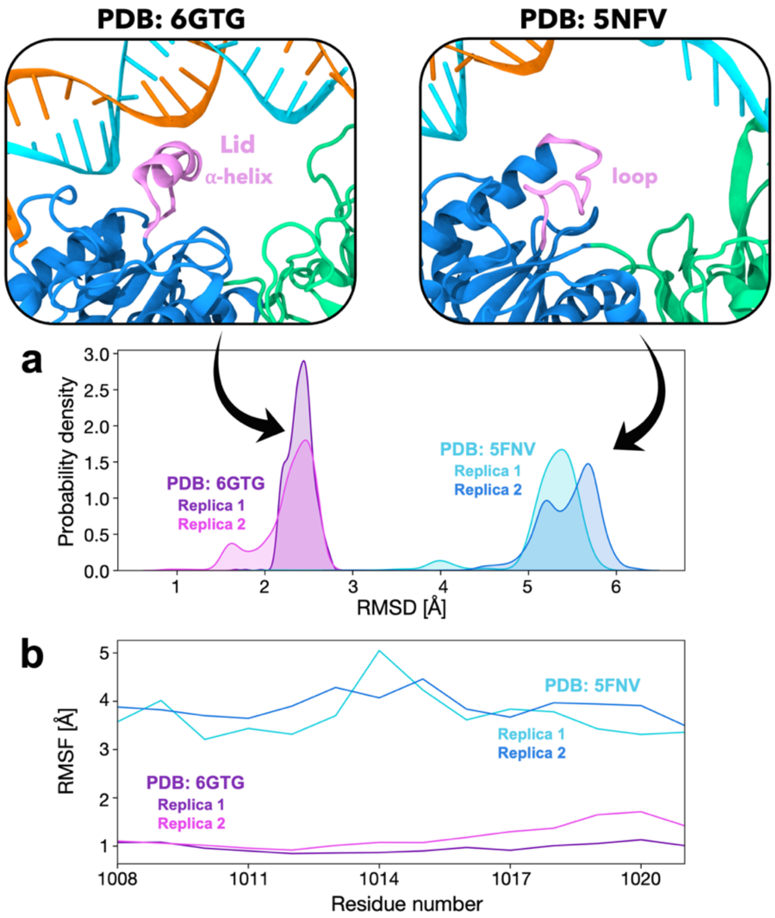

Supplementary Fig. 3: Stability (a) and flexibility (b) of the lid (residues L1008–K1021) along MD simulations. a Probability density plots of the all-atom root-mean-square deviation (RMSD) of the lid along MD simulations of the cryo-EM structure PDB: 6GTG [https://doi.org/10.2210/pdb6GTG/pdb] (Cas12a – I4 Conformation) of CRISPR-Cas12a including a complete TS (magenta) and of the X-ray structure PDB: 5NFV [https://doi.org/10.2210/pdb5NFV/pdb] (FnCas12a bound to R-loop) (blue), with respect to the structures obtained experimentally. Hydrogen atoms were excluded from the RMSD calculation. The 6GTG [https://doi.org/10.2210/pdb6GTG/pdb] (Cas12a – I4 Conformation) displays an α-helical lid (top panel, left), while the X-ray structure 5NFV [https://doi.org/10.2210/pdb5NFV/pdb] (FnCas12a bound to R-loop) reports an unstructured loop (top panel, right). Data are reported for two simulation replicates of ~10 μs each. **b** Root-mean-square fluctuations (RMSF) of the Cα atoms of the lid along MD simulations of the CRISPR-Cas12a systems defined above. Markedly higher RMSD and RMSF are observed for the lid assuming the shape of an unstructured loop as compared to the stable α-helical lid.

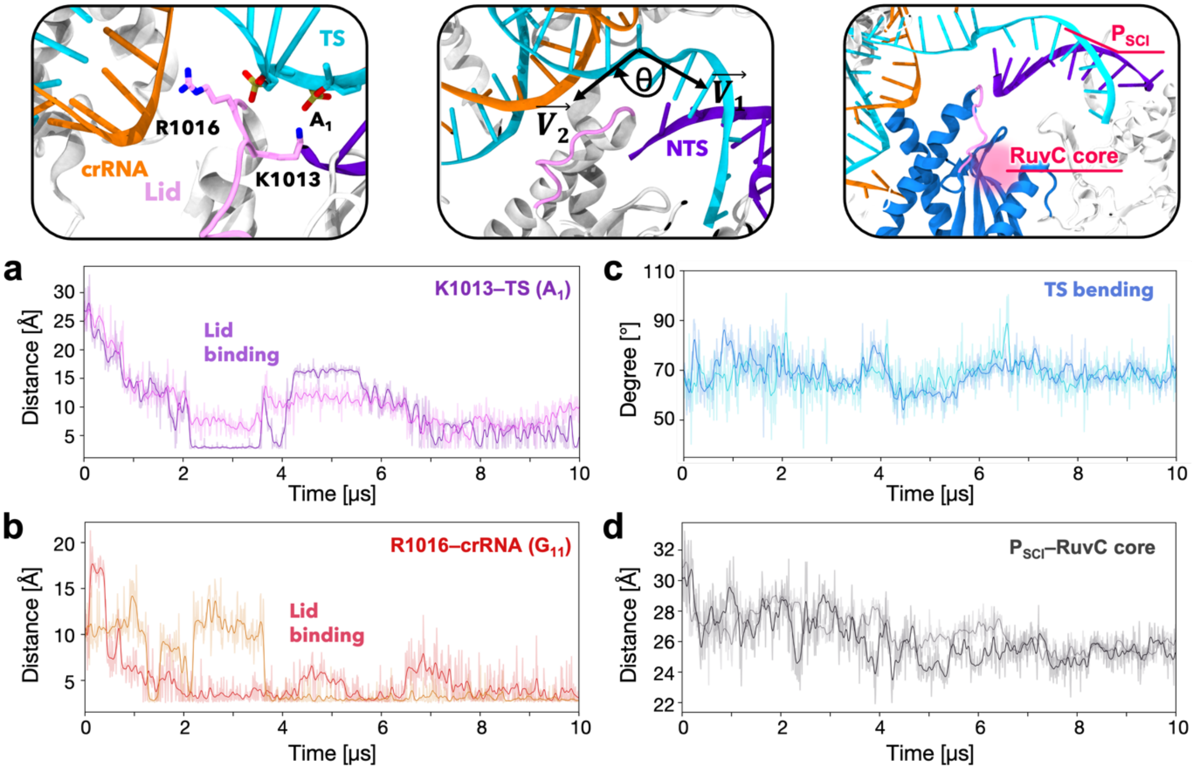

Supplementary Fig. 4: Molecular dynamics (MD) simulations of the 5NFV [https://doi.org/10.2210/pdb5NFV/pdb] (FnCas12a bound to R-loop) **X-ray structure of CRISPR-Cas12a (Fig. 1b).** Molecular simulations were extended to ~10 μs in two replicates. **a – b** Time evolution of the distances between positively charged residues of the unstructured lid and the crRNA:TS duplex (top panel, left). The top panel **(a)** reports the interaction between the lid and the TS (K1013:NZ – A_1:OP1), while the bottom panel **(b)** shows the interaction between the lid and the crRNA (R1016:NH1 – G_{11}:OP1). Interactions are measured as oxygen-nitrogen distance and are considered formed under 4 Å distance.[49] **c** Angle between the vectors used to represent the bending of the TS, as shown in the cartoon representation (top panel, middle) and explained in the Supplementary Methods. **d** Distance between the scissile phosphate (P_{SCI} between positions -2 and -3) in the DNA target strand (TS) and the RuvC catalytic pocket (D1255, D917, and E1006; top panel, right). The translucent lines in the plots show the actual data points, while the solid lines show the running average over 5 ns windows of MD simulation.

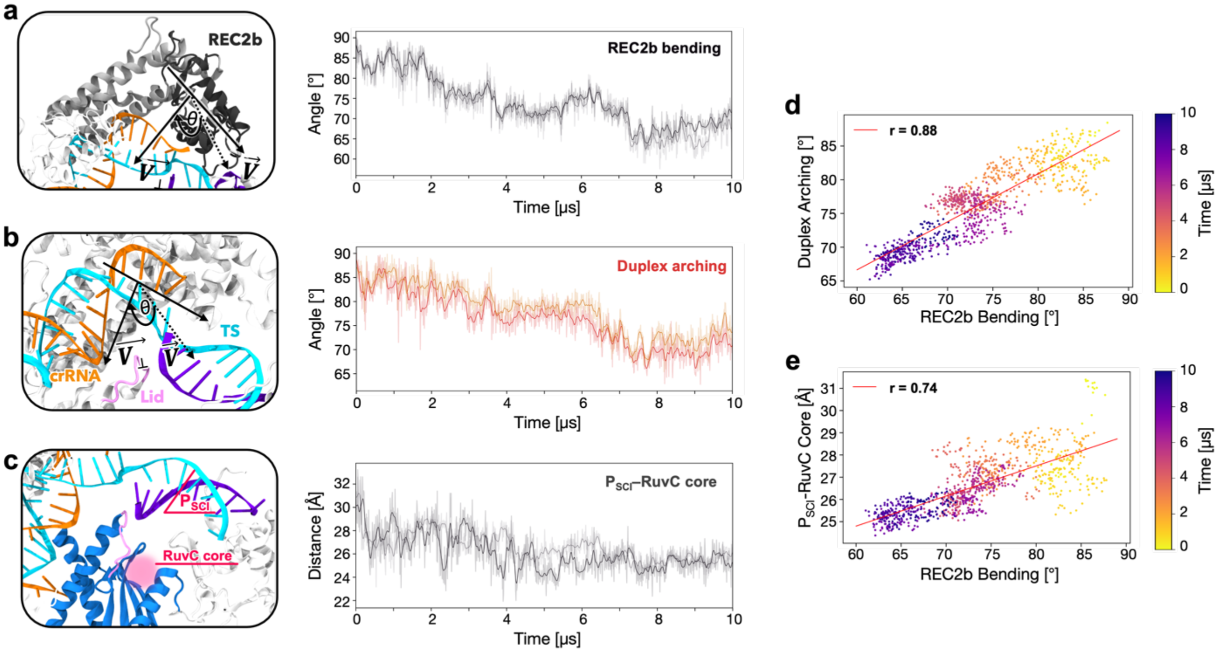

Supplementary Fig. 5: Bending of major domains and effects on the scissile phosphate along MD simulations of the 5NFV [https://doi.org/10.2210/pdb5NFV/pdb] (FnCas12a bound to R-loop) **X-ray structure of CRISPR-Cas12a (Fig. 1b). a – b** Time evolution of the bending angles describing **(a)** the bending of REC2b; **(b)** the arching of the terminal major groove of the crRNA:TS duplex. Angles between the vector passing through the region of interest and its perpendicular out of the plane were analysed with respect to the first frame of MD production runs. A representative snapshot of the system is reported on the left of each graph, showing the analysed angles (details in the Supplementary Methods). **c** Distance between the scissile phosphate (P_{SCI} between positions -2 and -3) in the DNA target strand (TS) and the RuvC catalytic pocket. The translucent lines in the plots show the actual data points, while the solid lines show the running average over 5 ns windows of MD simulation. **d – e** Scatter plots between **(d)** REC2b bending and duplex arching (**a – b**), where Pearson correlation coefficient, r = 0.88; and **(e)** REC2b bending and P_{SCI} – RuvC core distance (**a, c**), where Pearson correlation coefficient, r = 0.74. Data are reported for two simulation replicates of ~10 μs each and the correlation coefficient was calculated combining data from two replicates.

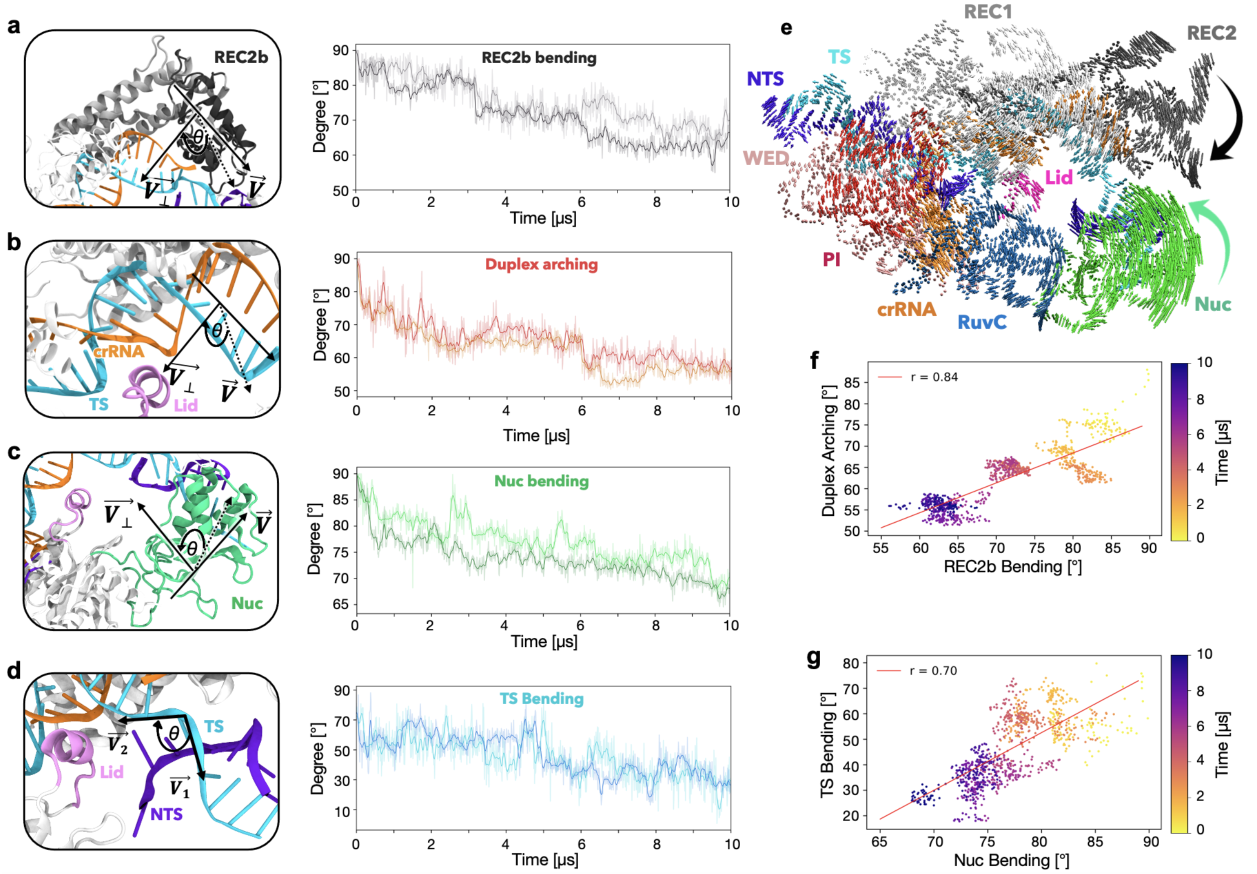

Supplementary Fig. 6: Bending of major domains along MD simulations of the complete CRISPR-Cas12a system (Fig. 1c). a – d Time evolution along MD simulations of bending angles describing **(a)** the bending of REC2b; **(b)** the arching of the terminal major groove of the crRNA:TS duplex; **(c)** the bending of Nuc and **(d)** the bending of the DNA TS region formed by bases G$_{-1}$, G$_1$, C$_2$, C$_3$ and T$_4$. Angles between the vector passing through the region of interest and its perpendicular out of the plane were analysed with respect to the first frame of MD production runs. A representative snapshot of the system is reported on the left of each graph, showing the analysed angles (details in the Supplementary Methods). The translucent lines in the plots show the actual data points, while the solid lines show the running average over 5 ns windows of MD simulation. **e** "Essential dynamics", derived from the first principal component (PC1) of the complete CRISPR-Cas12a system, shown using arrows of sizes proportional to the amplitude of motions. **f – g** Scatter plots between **(f)** REC2b bending and duplex arching (**a – b**), where Pearson correlation coefficient, r = 0.84; and **(g)** Nuc bending and TS bending (**c – d**), where Pearson correlation coefficient, r = 0.70. Data are reported for two simulation replicates of ~10 μs each and the correlation coefficient was calculated combining data from two replicates.

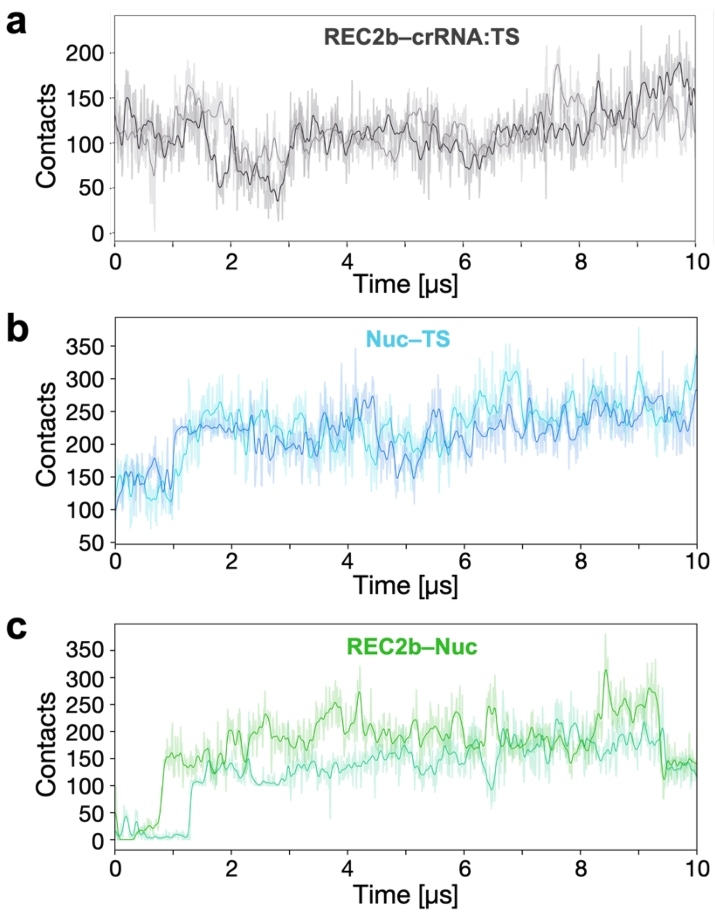

Supplementary Fig. 7: Number of contacts between major domains of Cas12a and nucleic acids. Molecular dynamics simulations of the complete CRISPR-Cas12a system show a significant number of contacts between: **(a)** the REC2b region and the crRNA:TS duplex; **(b)** the Nuc domain and the DNA target strand (TS); and **(c)** Rec2b and Nuc. Data are reported for two simulation replicates of ~10 μs each. A contact is considered when the distance between two heavy atoms among the regions of interest is less than 3.5 Å. The translucent lines in the plots show the actual data points, while the solid lines show the running average over 5 ns windows of MD simulation.

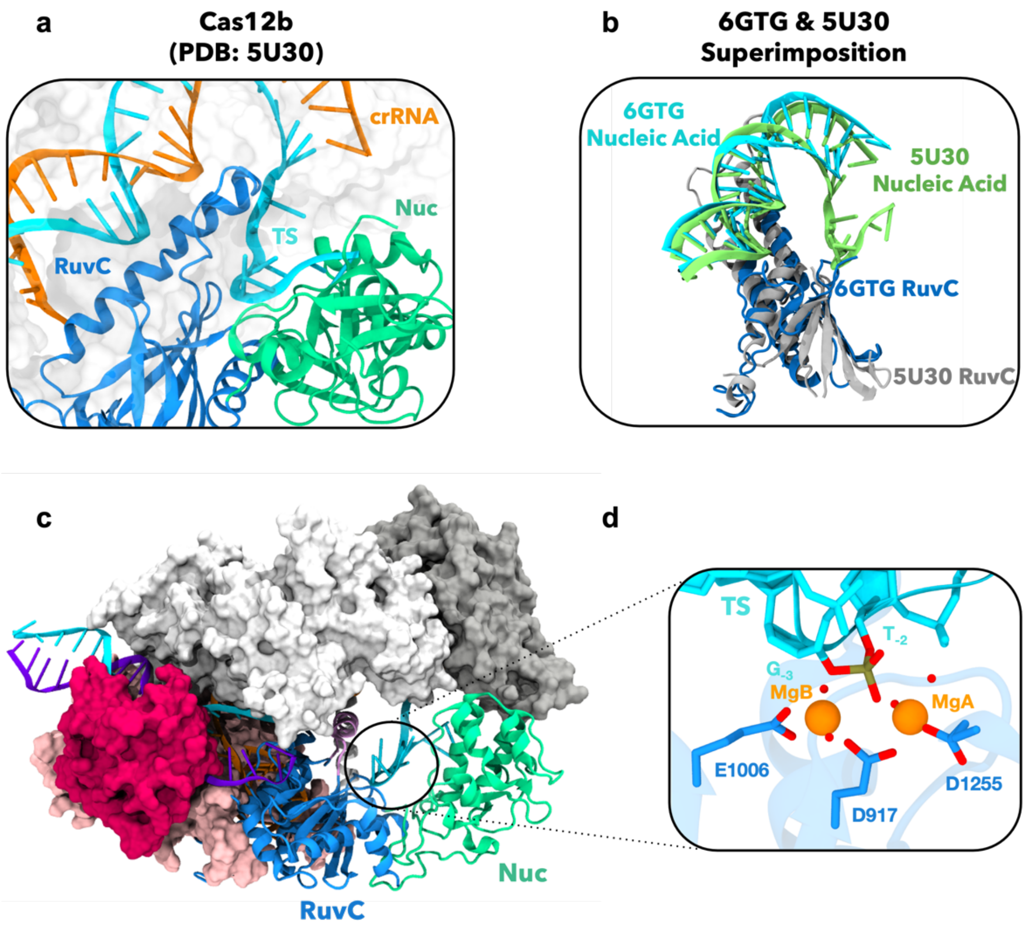

Supplementary Fig. 8: Overview of the catalytically competent CRISPR-Cas12a built from related X-ray structures. The protein is shown in molecular surface, highlighting the RuvC (blue) and Nuc (green) domains as ribbons. The crRNA (orange), the DNA target strand (TS, cyan) and non-target strand (NTS, violet) are also shown as ribbons. Mg^{2+} ions are shown as spheres. **a** X-ray structure of CRISPR-Cas12b (PDB: 5U30 [https://doi.org/10.2210/pdb5U30/pdb] (AacC2c1-sgRNA-extended target DNA ternary complex)),[11] showing the accommodation of the TS at the RuvC catalytic cleft. The TS bends down from the crRNA:TS duplex toward the active site, forming a U-shaped strand with its 5'-tail interacting with the Nuc domain. **b** Superimposition of the RuvC region and part of the nucleic acid from the cryo-EM structure (PDB: 6GTG [https://doi.org/10.2210/pdb6GTG/pdb] (Cas12a – I4 Conformation)) of Cas12a (protein in blue and nucleic acids in cyan) and the X-ray structure (PDB: 5U30 [https://doi.org/10.2210/pdb5U30/pdb] (AacC2c1-sgRNA-extended target DNA ternary complex)) of Cas12b (protein in gray and nucleic acid in gray) sharing structural homology. **c** Catalytically competent Cas12a for TS cleavage, including the TS in the RuvC catalytic cleft. The model was

built using the structures in (**a**) and (**b**) as references (details are reported in the SI text). **d** Close-up view of the RuvC active site, accommodating the TS in the two-metal ion architecture.

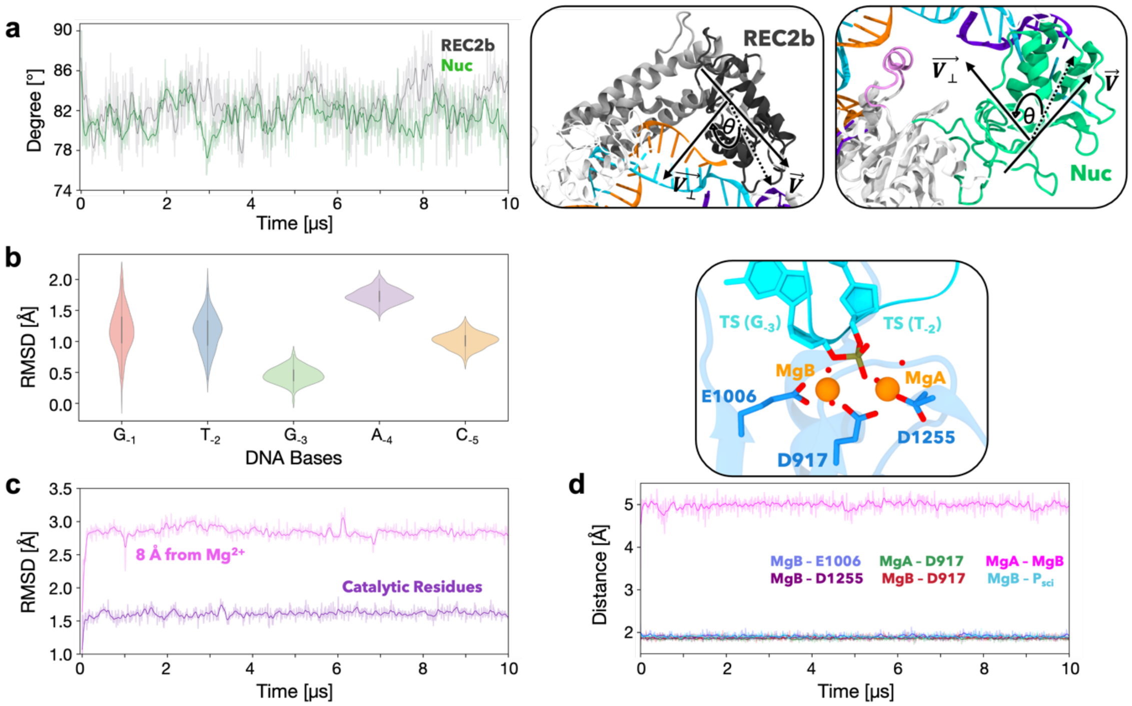

Supplementary Fig. 9: Molecular dynamics simulations of the catalytically competent CRISPR-Cas12a for TS cleavage. a Time evolution of the angles describing the bending of REC2b (gray) and Nuc (green) along ~10 μs long MD simulations. Angles between the vector passing through the region of interest and its perpendicular out of the plane were analysed with respect to the first frame of MD production runs. A representative snapshot on the right panel shows the analysed angles (details in the Supplementary Methods). This analysis shows that REC2b and Nuc assume a stable conformation. **b – d** Stability of the RuvC active site. **b** All-atom root-mean-square deviation (RMSD) of the DNA TS nucleobases close to the catalytic core. The RMSD of the nucleobases interacting with the catalytic Mg2+ ions involves T_{-2} (T_{-2}:OP1 – MgA; RMSD < 1.5 A) and G_{-3} (G_{-3}:OP3 – MgB; RMSD < 0.5 A), report remarkable stability. The flanking nucleobases also show appreciably low RMSD, highlighting the stability of the TS at the active site. **c** Time evolution of the RMSD of the catalytic residues around 8 Å of Mg^{2+} ions (pink), and the D917, E1006 and D1255 residues (violet). This analysis reveals that the RuvC catalytic site with the DNA TS is remarkably stable along ~10 μs of MD simulations. **d** Distance analyses between the key catalytic residues (D917, E1006 and D1255) and the two catalytic Mg^{2+} ions (MgA and MgB) evolving over ~10 μs MD simulation, exhibiting noteworthy stability of the catalytic core. A representative snapshot of the RuvC catalytic core with the DNA TS is shown above the graph. The translucent lines in the plots show the actual data points, while the solid lines show the running average over 5 ns windows of MD simulation.

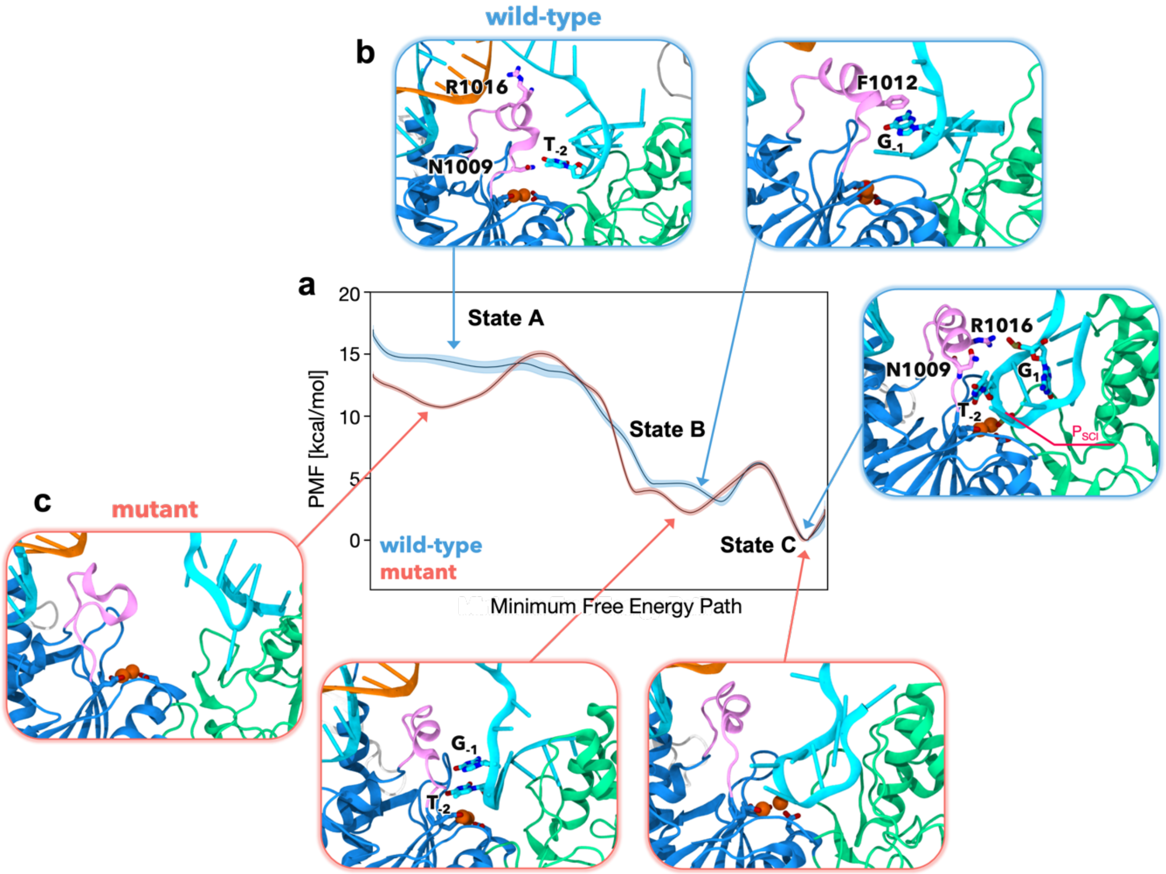

Supplementary Fig. 10: Free energy profiles for the traversal of the DNA target strand (TS) toward the RuvC catalytic core. a Free energy profiles for the wild-type (WT) Cas12a (blue) and upon mutating relevant residues of the α-helical lid (F1012, N1009, K1013, R1014, R1016, K1018) into alanine (red). The Potential of Mean Force (PMF) is computed from 2-D umbrella sampling simulations and plotted along the minimum free energy path (see the Supplementary Methods). Representative snapshots, indicated by arrows, are based on the reaction coordinate values along the minimum free energy path of the wild-type Cas12a **(b)** and its mutant **(c)**.

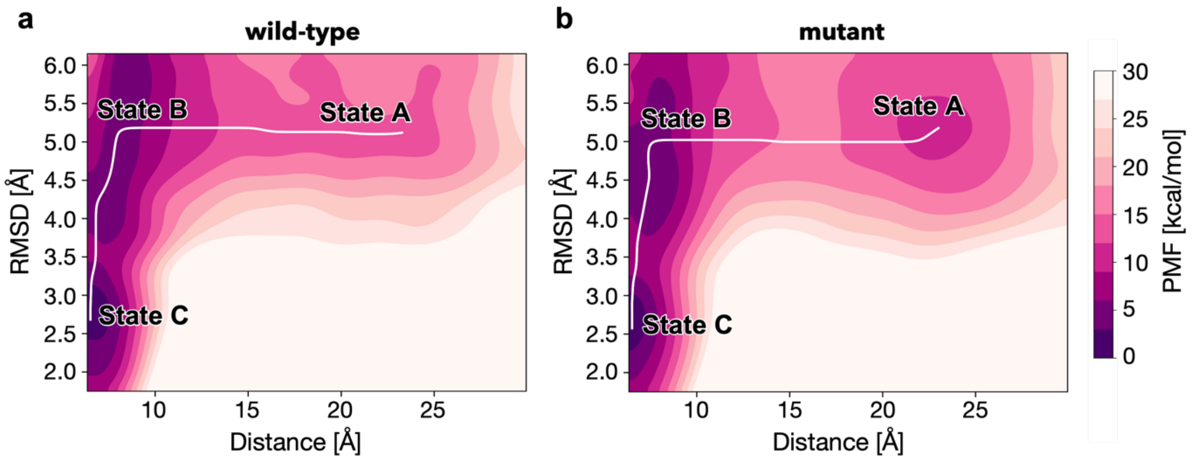

Supplementary Fig. 11: Two-dimensional (2-D) free energy maps for the traversal of the DNA target strand (TS) toward the RuvC core. a – b 2-D Umbrella Sampling simulations for the wild-type Cas12a **(a)** and upon mutating critical residues of the α-helical lid (F1012, N1009, K1013, R1014, R1016, K1018) into alanine **(b)**. The Potential of Mean Force (PMF) is computed along two Reaction Coordinates (RCs). RC1 is the root-mean-square deviation (RMSD) of the Cα atoms between the initial and the final state. RC2 is the distance between the centre of mass of the RuvC catalytic core (D917, E1006, D1255) and the TS region including the scissile phosphate (i.e., the DNA nucleobases at positions -2 to -4, as indicated by biochemical and single-molecule experiments). Full details are reported in the Supplementary Methods. The solid white line represents the minimum free energy path connecting the initial and final states. The different states along the minimum free energy path are indicated (also in Supplementary Fig. 8).

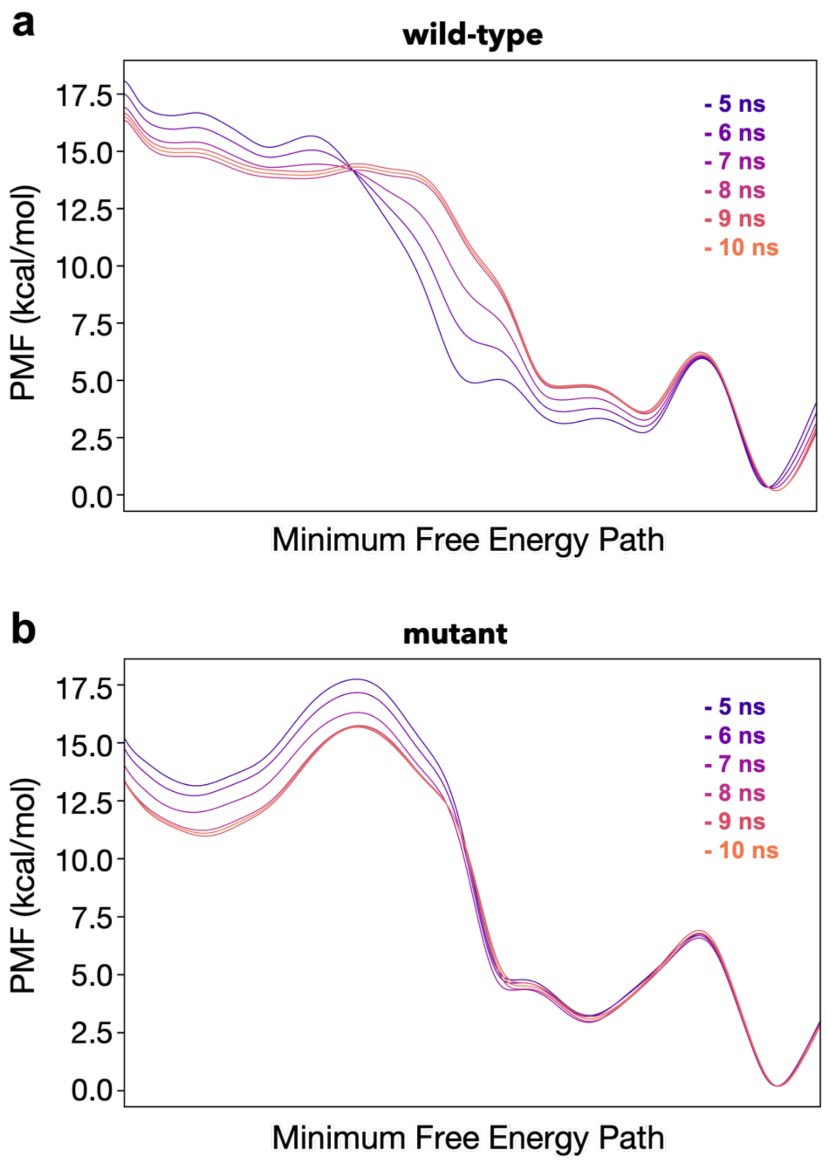

Supplementary Fig. 12: Convergence of the free energy profiles obtained through Umbrella Sampling (US) simulations. a – b Convergence of the potential of mean force (PMF) along the minimum free energy path for US simulations of the wild-type CRISPR-Cas12a **(a)**, and including alanine mutations of critical residues of the α-helical lid **(b)**. N1009, F1012, K1013, R1014, R1016 and K1018 were mutated into alanine to probe their role in the traversal of the DNA target strand. The PMF (kcal/mol) was computed for sampling until 5 ns, 6 ns, 7 ns, 8 ns, 9 ns and 10 ns after removing the biases incurred by the restraints during the sampling. Convergence is reached ~8 ns onward for both the systems and we used bin sizes of ~10 ns to compute our 2-D free energy surfaces (**Supplementary Fig. 9**) and the minimum free energy paths (**Supplementary Fig. 10** and Fig. 4).

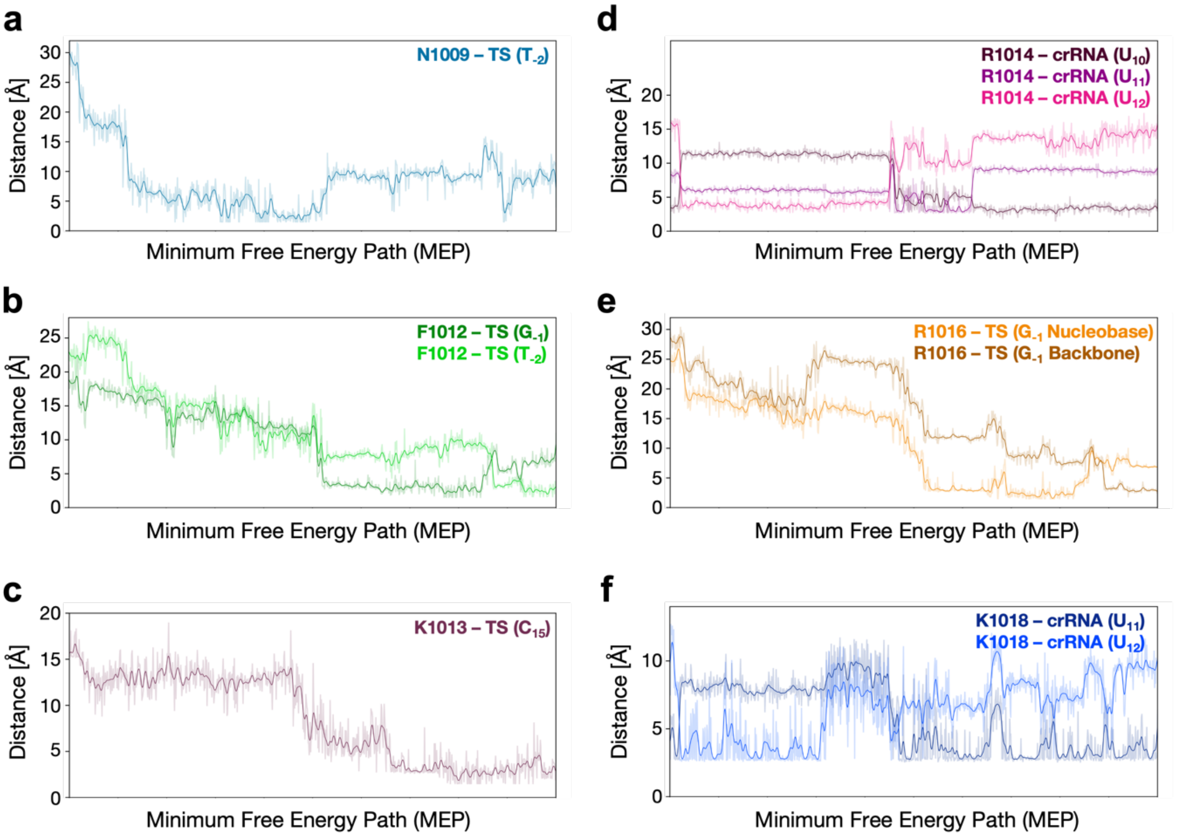

Supplementary Fig. 13: Interactions between the lid and the crRNA:TS duplex along the minimum energy path (MEP) from Umbrella Sampling simulations of the wild-type CRISPR-Cas12a. a – f Distances between **(a)** N1009 – TS (N1009:ND2 – T_{-2}:OP1), **(b)** F1012 – TS (F1012:phenylalanine ring – G_{-1}:imidazole ring and F1012:phenylalanine ring – T_{-2}:pyrimidine ring), **(c)** K1013 – TS (K1013:KZ – C_{15}:OP1), **(d)** R1014 – crRNA (R1014:NH2 – U_{10}:OP1, R1014:NH2 – U_{11}:OP1 and R1014:NH2 – U_{12}:OP1), **(e)** R1016 – TS (R1016:NH2 – G_{-1}:nucleobase and G_{-1}:OP1), and **(f)** K1018 – crRNA (K1018:NZ – U_{11}:OP1 and K1018:NZ – U_{12}:OP1). The translucent lines show the actual data points, while the solid lines show the running average over 5 ns windows of MD simulation. The free energy profiles along the MEP are reported in Fig. 4a and Supplementary Fig. 10.

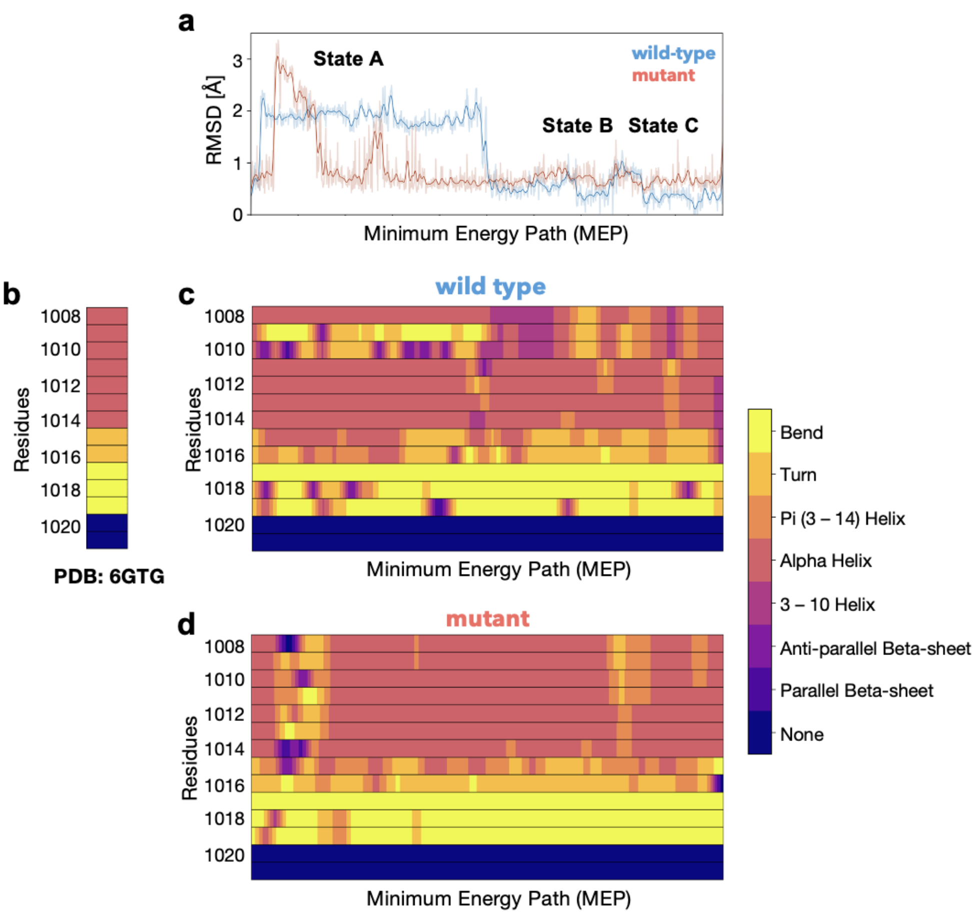

Supplementary Fig. 14: Lid dynamics along the minimum energy path (MEP) of the Umbrella sampling simulation. a Root-mean-square deviation (RMSD) of the lid (L1008 – K1021) along the minimum free energy path of the Umbrella sampling simulations (Fig. 4 and Supplementary Fig. 10) for the wild-type (blue) and mutant (red) systems. The translucent lines in the plots show the actual data points, while the solid lines show the running average over 5 ns windows of MD simulation. **b – d** Secondary structure analysis of the lid residues using the DSSP algorithm[50,51] for **(b)** the cryo-EM Cas12a structure (PDB: 6GTG [https://doi.org/10.2210/pdb6GTG/pdb] (Cas12a – I4 Conformation)), **(c)** wild-type, and **(d)** mutant systems. The secondary structure analysis for the wild-type and mutant systems were performed along the minimum free energy path of the Umbrella sampling simulations (Supplementary Fig. 10 and 11).

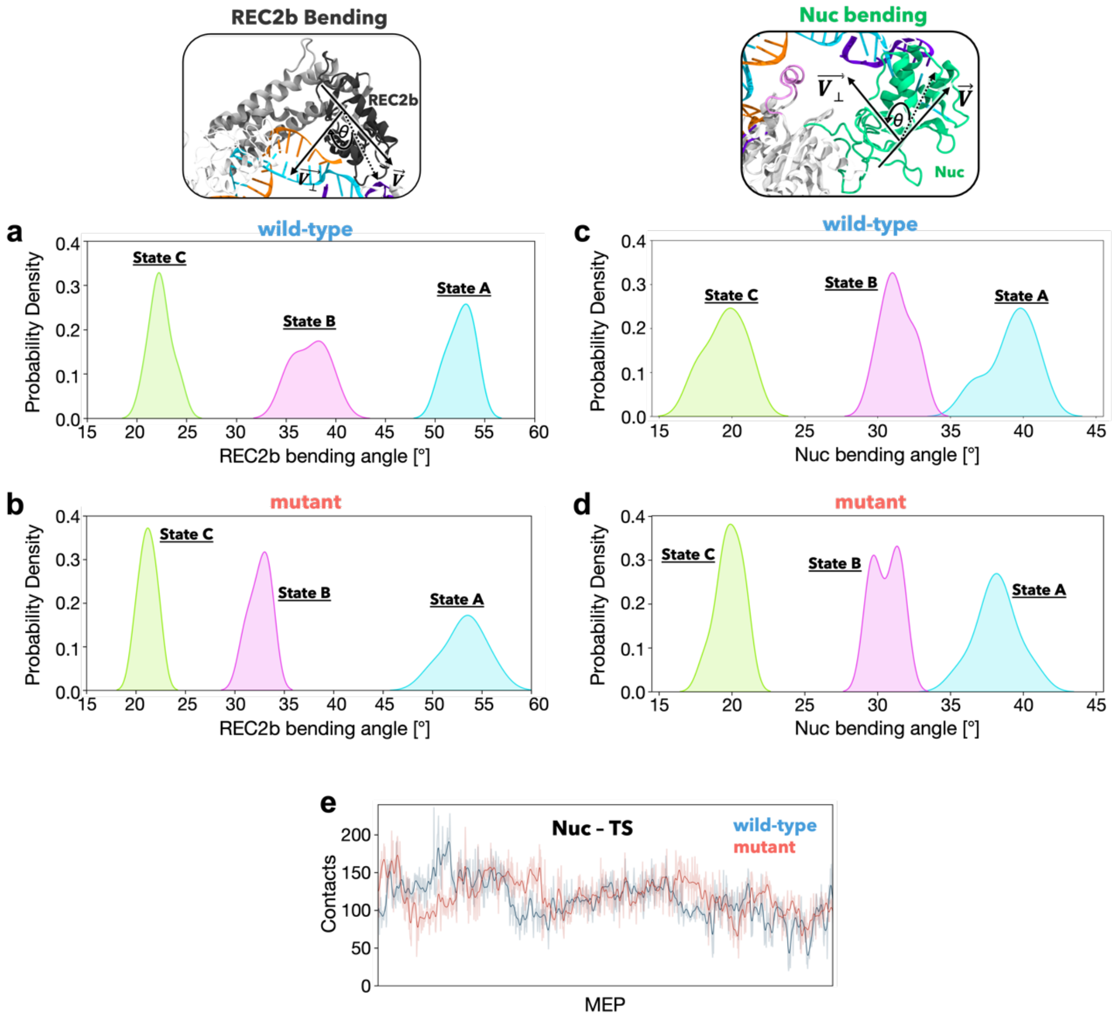

Supplementary Fig. 15: Dynamics of major domains at different states along the free energy profiles of the Umbrella sampling simulations. a – d Bending angle of the REC2b region and Nuc domain is computed at three different states (states A-C; Supplementary Fig. 10 and 11) along the minimum energy path (MEP) of the umbrella sampling simulations. Bending of **(a – b)** REC2b and **(c – d)** Nuc of the wild-type Cas12a and its mutant. Moving from state A to C, REC2b and Nuc moves inward, as indicated by a decrease of its bending angle in the representative snapshots. **e** Contacts between Nuc and TS along the MEP. A contact is considered when the distance between two heavy atoms among the regions of interest is less than 3.5 Å. The translucent lines in the plots show the actual data points, while the solid lines show the running average over 5 ns windows of MD simulation.

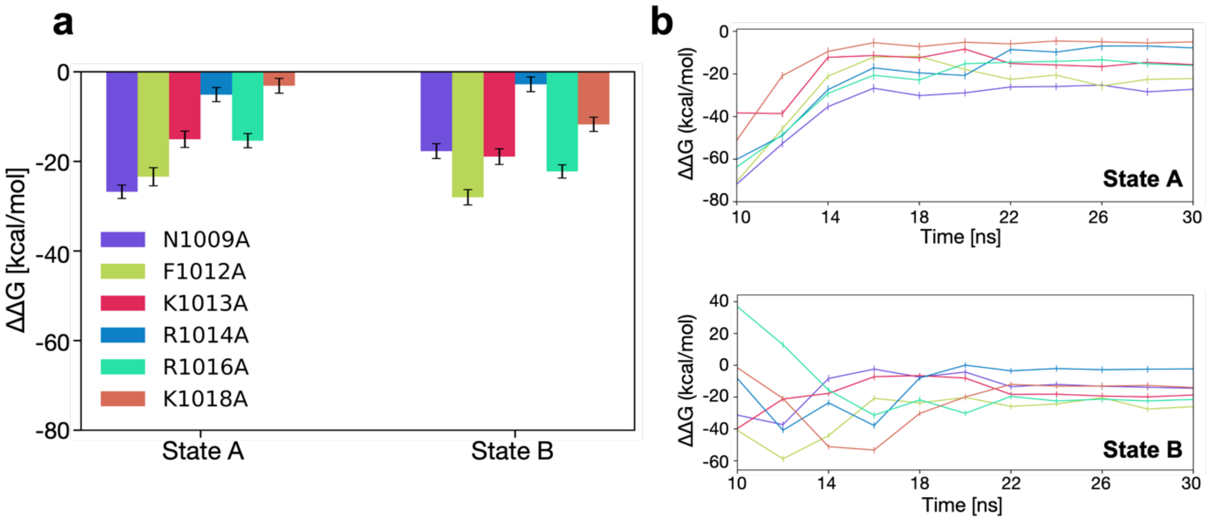

Supplementary Fig. 16: Convergence of the relative free energy changes obtained through alchemical free energy simulations (AFE). a Relative free energy changes (ΔΔG, in kcal/mol) of the individual residues with respect to alanine in binding the DNA TS at both the states encompassing the traversal of the TS guided by the α-helical lid, i.e., states A and B. Binding free energies are computed through the Multistate Bennett Acceptance Ratio (MBAR) method[43,44] (see the Methods section) and denoted with the associated MBAR error. **b** Convergence profile of the relative binding free energies computed until 10 ns, 12 ns, 14 ns, 16 ns, 18 ns, 20 ns, 22 ns, 24 ns, 26 ns, 28 ns and 30 ns of the alchemical free energy simulations are shown with the MBAR error. Convergence is reached ~22 ns onward for both states A and B.

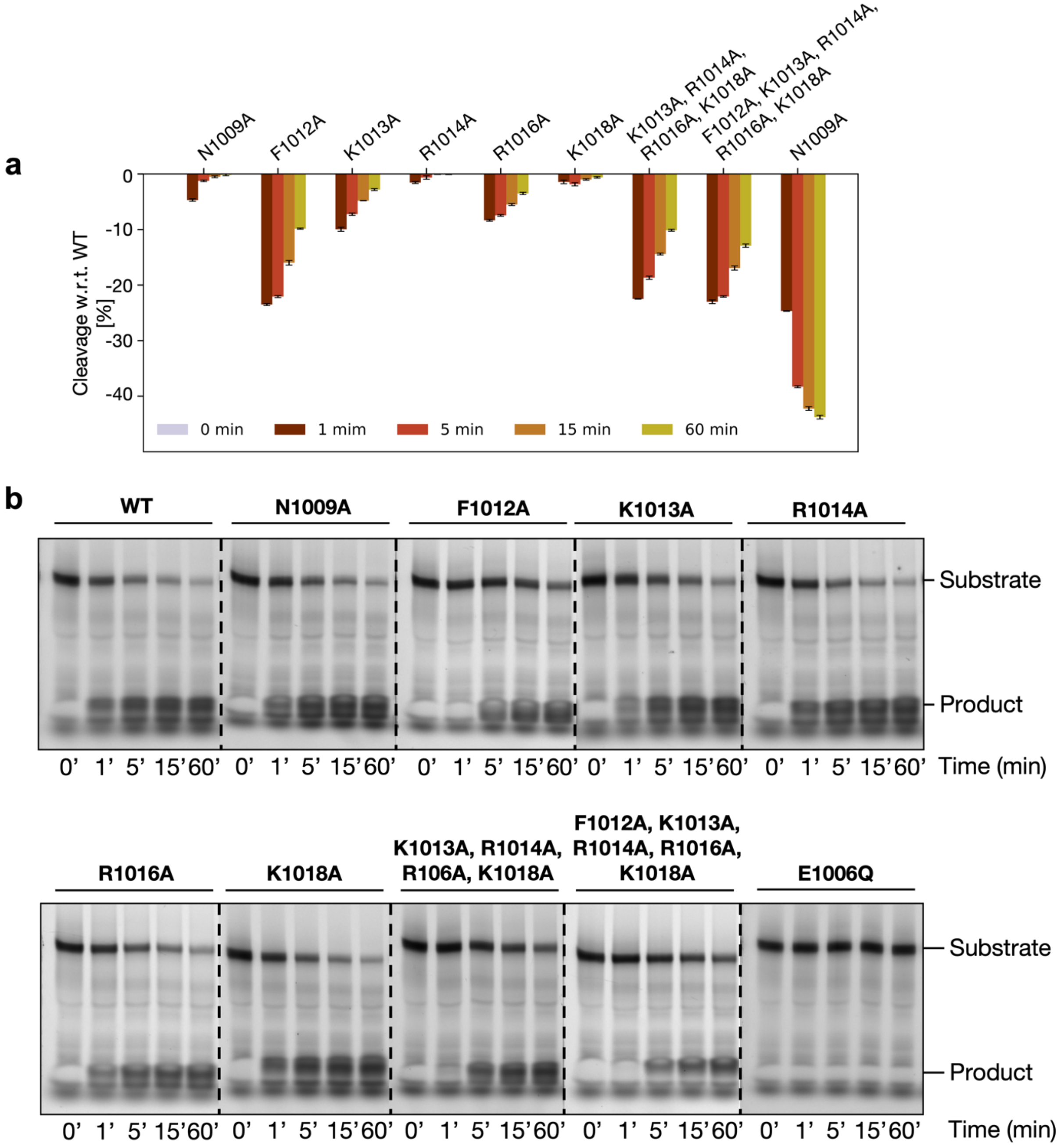

Supplementary Fig. 17: *In vitro* DNA TS cleavage assay of Cas12a mutants. a DNA target strand (TS) cleavage percentage of Cas12a mutants compared to the wild type (WT). Data represents n = 3 independent replicates and the mean is plotted with the standard error of mean. **b** Denaturing polyacrylamide gels showing DNA TS cleaved products by Cas12a mutants at 0 min, 1 min, 5 min, 15 min and 60 min. Replica 1 from the three independent replicates has been shown here for each of the mutants (Supplementary Fig. 18, labelled with an asterisk sign).

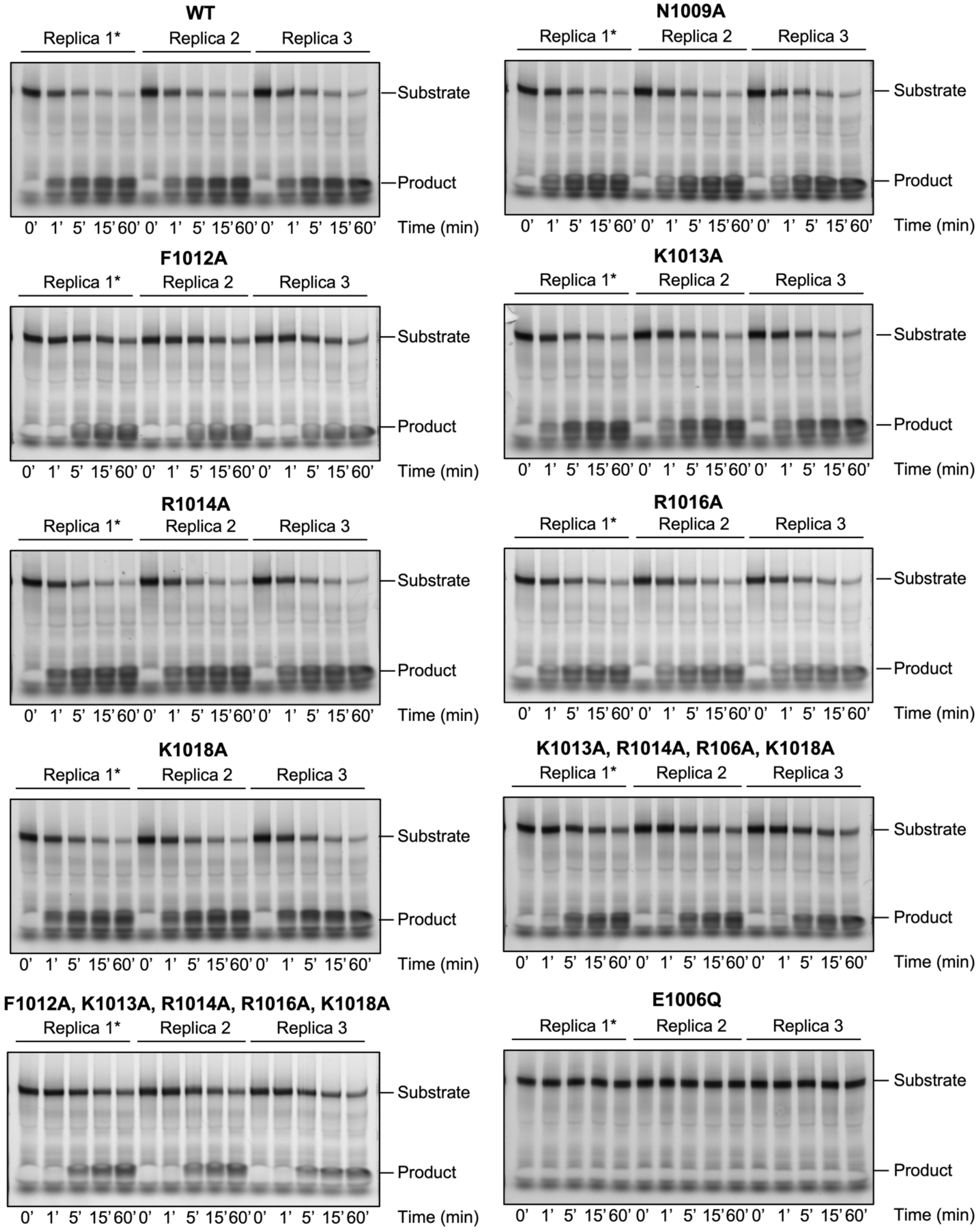

Supplementary Fig. 18: *In vitro* dsDNA cleavage assay of Cas12a mutants. Cropped denaturing polyacrylamide gels showing DNA TS cleaved products by Cas12a mutants at 0 min,

1 min, 5 min, 15 min and 60 min. Data represents n = 3 independent replicates. Replica 1 has been labelled with an asterisk sign to indicate the replica shown in Supplementary Fig. 13.

Supplementary Tables

Supplementary Table 1: Description of the simulated complexes.

Sl. No.	Name	Salient Features
1	Cryo-EM Cas12a	PDB ID: 6GTG [https://doi.org/10.2210/pdb6GTG/pdb] (Cas12a – I4 Conformation) (Stella et al. *Cell* **2018**, 165, 1856-1871). Contains α-helical lid (L1008 – K1021).
2	X-ray Cas12a	PDB ID: 5NFV [https://doi.org/10.2210/pdb5NFV/pdb] (FnCas12a bound to R-loop) (Swarts et al. *Mol. Cell* **2017**, 66, 221-233). Contains an unstructured loop form of the lid as reconstructed using Homology Modelling (details in the Supplementary Methods).
3	Complete Cas12a	Based on PDB ID: 6GTG [https://doi.org/10.2210/pdb6GTG/pdb] (Cas12a – I4 Conformation) and including a longer TS downstream of the crRNA:TS duplex, taking cues from PDB ID: 5NFV [https://doi.org/10.2210/pdb5NFV/pdb] (FnCas12a bound to R-loop) (details in the Supplementary Methods).
4	Catalytically Competent Cas12a	Based on the complete Cas12a system and PDB: 5U30 [https://doi.org/10.2210/pdb5U30/pdb] (AacC2c1-sgRNA-extended target DNA ternary complex) (Yang et al. *Cell* **2016**, 167, 1814-1828). The 5'-tail of the TS downstream of the crRNA:TS duplex, which includes the scissile phosphate, locates in the RuvC active (details in the Supplementary Methods).

Supplementary Table 2. Details for the calculation of bending angles using vectors. For each bending angle, we selected two regions of interest (mask 1 and mask 2), used as a reference to compute the vectors ($\vec{V}$) for angle analysis. Details are in the Supplementary Methods.

**Bending angle**	**Mask 1**	**Mask 2**
crRNA:TS arching	crRNA (A_{16}):TS (T_5)	crRNA (G_{19}):TS (C2)
TS bending	Nucleobases C_2 – G_{-1}	Nucleobases G_{-1} – G_{-3}
Rec2 bending	Residues I370 – K382	Residues L451 – D470
Nuc bending	Residues H1187 – C1196	Residues F1240 – M1250

Supplementary Table 3. Sequences of crRNA guide and target DNA substrates

ID	Description	Sequence (5'-3')
oMS017	FnCas12a-crRNA⌊	AAUUUCUACUGUUGUAGAUGUGAUAAGUGGAAUGCCAUGUGGG
oDS271	NTS⌊	ACGTGGAAGGATGCCTTTAGTGATAAGTGGAATGCCATGTGGGCTGTC AAAATTGAGT
oDS285	labeled TS⌊	(ATTO532-)ACTCAATTTTGACAGCCCACATGGCATTCCACTTATCACTAAAGGCATCCTTCCACGT

Supplementary References

1. Stella, S. *et al.* Conformational Activation Promotes CRISPR-Cas12a Catalysis and Resetting of the Endonuclease Activity. *Cell* **175**, 1856–1871 (2018).

2. Swarts, D. C., van der Oost, J. & Jinek, M. Structural Basis for Guide RNA Processing and Seed-Dependent DNA Targeting by CRISPR-Cas12a. *Mol. Cell* **66**, 221–233 (2017).

3. Biasini, M. *et al.* SWISS-MODEL: modelling protein tertiary and quaternary structure using evolutionary information. *Nucleic Acids Res.* **42**, 252–258 (2014).

4. Waterhouse, A. *et al.* SWISS-MODEL: homology modelling of protein structures and complexes. *Nucleic Acids Res.* **46**, W296–W303 (2018).

5. Cofsky, J. C. *et al.* CRISPR-Cas12a exploits R-loop asymmetry to form double-strand breaks. *Elife* **9**, e55143 (2020).

6. Fiser, A. & Šali, A. Modeller: Generation and Refinement of Homology-Based Protein Structure Models. *Methods Enzymol.* **374**, 461–491 (2003).

7. Yang, W. Nucleases: diversity of structure, function and mechanism. *Q. Rev. Biophys.* **44**, 1–93 (2011).

8. Bravo, J. P. K. *et al.* Structural basis for mismatch surveillance by CRISPR–Cas9. *Nature* **603**, 343–347 (2022).

9. Jinek, M. *et al.* A Programmable Dual-RNA-Guided DNA Endonuclease in Adaptive Bacterial Immunity. *Science* **337**, 816–821 (2012).

10. Huang, X. *et al.* Structural basis for two metal-ion catalysis of DNA cleavage by Cas12i2. *Nat. Commun.* **11**, 5241 (2020).

11. Yang, H., Gao, P., Rajashankar, K. R. & Patel, D. J. PAM-Dependent Target DNA Recognition and Cleavage by C2c1 CRISPR-Cas Endonuclease. *Cell* **167**, 1814–1828 (2016).

12. Shaw, D. E. *et al.* Anton 2: Raising the Bar for Performance and Programmability in a Special-Purpose Molecular Dynamics Supercomputer. In *SC14: International Conference for High Performance Computing, Networking, Storage and Analysis* 41–53 (IEEE, 2014).

13. Tian, C. *et al.* ff19SB: Amino-Acid-Specific Protein Backbone Parameters Trained against Quantum Mechanics Energy Surfaces in Solution. *J. Chem. Theory Comput.* **16**, 528–552 (2020).

14. Ivani, I. *et al.* Parmbsc1: a refined force field for DNA simulations. *Nat. Methods* **13**, 55–58 (2016).

15. Banas, P. *et al.* Performance of Molecular Mechanics Force Fields for RNA Simulations: Stability of UUCG and GNRA Hairpins. *J. Chem. Theor. Comput.* **6**, 3836–3849 (2010).

16. Zgarbova, M. *et al.* Refinement of the Cornell et al. Nucleic Acids Force Field Based on Reference Quantum Chemical Calculations of Glycosidic Torsion Profiles. *J. Chem. Theory Comput.* **7**, 2886–2902 (2011).

17. Jorgensen, W. L., Chandrasekhar, J., Madura, J. D., Impey, R. W. & Klein, M. L. Comparison of simple potential functions for simulating liquid water. *J. Chem. Phys.* **79**, 926–935 (1983).

18. Li, P., Roberts, B. P., Chakravorty, D. K. & Merz, K. M. Rational Design of Particle Mesh Ewald Compatible Lennard-Jones Parameters for +2 Metal Cations in Explicit Solvent. *J. Chem. Theory Comput.* **9**, 2733–2748 (2013).

19. Saha, A., Arantes, P. R. & Palermo, G. Dynamics and mechanisms of CRISPR-Cas9 through the lens of computational methods. *Curr. Opin. Struct. Biol.* **75**, 102400 (2022).

20. Palermo, G. *et al.* Key role of the REC lobe during CRISPR–Cas9 activation by ‘sensing’, ‘regulating’, and ‘locking’ the catalytic HNH domain. *Q. Rev. Biophys.* **51**, e9 (2018).

21. Nierzwicki, L. *et al.* Principles of target DNA cleavage and the role of Mg2+ in the catalysis of CRISPR–Cas9. *Nature Catalysis* **5**, 912–922 (2022).

22. Turq, P., Lantelme, F. & Friedman, H. L. Brownian Dynamics: Its Applications to Ionic Solutions. *J. Chem. Phys.* **66**, 3039–3044 (1977).

23. Berendsen, H. J. C., Postma, J. P. M., van Gunsteren, W. F., DiNola, A. & Haak, J. R. Molecular Dynamics with Coupling to an External Bath. *J. Chem. Phys.* **81**, 3684–3690 (1984).

24. Case, D. A. *et al.* AMBER 2020. *Univ. California, San Fr.* (2020).

25. Tuckerman, M., Berne, B. J. & Martyna, G. J. Reversible multiple time scale molecular dynamics. *J. Chem. Phys.* **97**, 1990–2001 (1992).

26. Lippert, R. A. *et al.* Accurate and efficient integration for molecular dynamics simulations at constant temperature and pressure. *J. Chem. Phys.* **139**, 164106 (2013).

27. Martyna, G. J., Tobias, D. J. & Klein, M. L. Constant pressure molecular dynamics algorithms. *J. Chem. Phys.* **101**, 4177–4189 (1994).

28. Hoover, W. G. Canonical dynamics: Equilibrium phase-space distributions. *Phys. Rev. A* **31**, 1695–1697 (1985).

29. Nosé, S. An extension of the canonical ensemble molecular dynamics method. *Mol. Phys.* **57**, 187–191 (1986).

30. Shan, Y., Klepeis, J. L., Eastwood, M. P., Dror, R. O. & Shaw, D. E. Gaussian split Ewald: A fast Ewald mesh method for molecular simulation. *J. Chem. Phys.* **122**, 054101 (2005).

31. Ryckaert, J.-P., Ciccotti, G. & Berendsen, H. J. . Numerical integration of the cartesian equations of motion of a system with constraints: molecular dynamics of n-alkanes. *J. Comput. Phys.* **23**, 327–341 (1977).

32. Amadei, A., Linssen, A. B. M. & Berendsen, H. J. C. Essential dynamics of proteins. *Proteins Struct. Funct. Genet.* **17**, 412–425 (1993).

33. Daidone, I. & Amadei, A. Essential dynamics: foundation and applications. *Wiley Interdiscip. Rev. Comput. Mol. Sci.* **2**, 762–770 (2012).

34. Bakan, A., Meireles, L. M. & Bahar, I. ProDy: Protein Dynamics Inferred from Theory and Experiments. *Bioinformatics* **27**, 1575–1577 (2011).

35. Humphrey, W., Dalke, A. & Schulten, K. VMD: visual molecular dynamics. *J Mol Graph* **14**, 27–28 (1996).

36. Kästner, J. Umbrella sampling. *Wiley Interdisciplinary Reviews: Computational Molecular Science* **1**, 932–942 (2011).

37. Naqvi, M. M., Lee, L., Montaguth, O. E. T., Diffin, F. M. & Szczelkun, M. D. CRISPR–Cas12a-mediated DNA clamping triggers target-strand cleavage. *Nat. Chem. Biol.* **18**, 1014–1022 (2022).

38. Kumar, S., Rosenberg, J. M., Bouzida, D., Swendsen, R. H. & Kollman, P. A. The weighted histogram analysis method for free-energy calculations on biomolecules. I. The method. *J. Comput. Chem.* **13**, 1011–1021 (1992).

39. Marcos-Alcalde, I., Setoain, J., Mendieta-Moreno, J. I., Mendieta, J. & Gomez-Puertas, P. MEPSA: minimum energy pathway analysis for energy landscapes. *Bioinformatics* **31**, 3853–3855 (2015).

40. Hub, J. S., de Groot, B. L. & van der Spoel, D. g_wham—A Free Weighted Histogram Analysis Implementation Including Robust Error and Autocorrelation Estimates. *J. Chem. Theory Comput.* **6**, 3713–3720 (2010).

41. Lee, T.-S. *et al.* Alchemical Binding Free Energy Calculations in AMBER20: Advances and Best Practices for Drug Discovery. *J. Chem. Inf. Model.* **60**, 5595–5623 (2020).

42. Lee, T.-S. *et al.* Improved Alchemical Free Energy Calculations with Optimized Smoothstep Softcore Potentials. *J. Chem. Theory Comput.* **16**, 5512–5525 (2020).

43. Shirts, M. R. & Chodera, J. D. Statistically optimal analysis of samples from multiple equilibrium states. *J. Chem. Phys.* **129**, 124105 (2008).

44. Matsunaga, Y. *et al.* Use of multistate Bennett acceptance ratio method for free-energy calculations from enhanced sampling and free-energy perturbation. *Biophys. Rev.* **14**, 1503–1512 (2022).

45. Klimovich, P. V., Shirts, M. R. & Mobley, D. L. Guidelines for the analysis of free energy calculations. *J. Computer-aided Mol. design* **29**, 397–411 (2015).

46. Wille, D., Jaeger, H., Schlaich, A., Wu, Z., & Beckstein, O. Flamel (2022). San Francisco (CA): GitHub. https://github.com/alchemistry/flamel.

47. Mohanraju, P., Oost, J., Jinek, M. & Swarts, D. Heterologous Expression and Purification of the CRISPR-Cas12a/Cpf1 Protein. *Bio-protocol* **8**, e2842 (2018).

48. Kissling, L., Monfort, A., Swarts, D. C., Wutz, A. & Jinek, M. Preparation and electroporation of Cas12a/Cpf1-guide RNA complexes for introducing large gene deletions in mouse embryonic stem cells. *Methods in Enzymology* **616**, 241–263 (2019).

49. Kumar, S. & Nussinov, R. Relationship between Ion Pair Geometries and Electrostatic

Strengths in Proteins. *Biophys. J.* **83**, 1595–1612 (2002).

50. Touw, W. G. *et al.* A series of PDB-related databanks for everyday needs. *Nucleic Acids Res.* **43**, 364–368 (2015).

51. Kabsch, W. & Sander, C. Dictionary of protein secondary structure: Pattern recognition of hydrogen-bonded and geometrical features. *Biopolymers* **22**, 2577–2637 (1983).
