# Supplementary material for: An alpha-helical lid guides the target DNA toward catalysis in CRISPR-Cas12a
Source: Nat Commun. 2024 Feb 17;15:1473. doi: 10.1038/s41467-024-45762-6 (PMC10874386; doi:10.1038/s41467-024-45762-6)
Supplement: Supplementary file 3 — Description of Additional Supplementary Files [file 41467_2024_45762_MOESM3_ESM.pdf]

Description of Additional Supplementary Files:

Supplementary Data 1: Sequences of DNA oligos, synthetic gene and gene fragments (gBlocks).

Supplementary Data 2: In vitro DNA cleavage assay time points data of Cas12a wild type and its mutants.
