# Supplementary material for: An alpha-helical lid guides the target DNA toward catalysis in CRISPR-Cas12a
Source: Nat Commun. 2024 Feb 17;15:1473. doi: 10.1038/s41467-024-45762-6 (PMC10874386; doi:10.1038/s41467-024-45762-6)
Supplement: Supplementary file 6 — Reporting Summary [file 41467_2024_45762_MOESM6_ESM.pdf]

nature portfolio

Corresponding author(s): Giulia Palermo
Last updated by author(s): Dec 1, 2023

Reporting Summary

Nature Portfolio wishes to improve the reproducibility of the work that we publish. This form provides structure for consistency and transparency in reporting. For further information on Nature Portfolio policies, see our Editorial Policies and the Editorial Policy Checklist.

Statistics

For all statistical analyses, confirm that the following items are present in the figure legend, table legend, main text, or Methods section.

n/a	Confirmed	
☐	☒	The exact sample size (*n*) for each experimental group/condition, given as a discrete number and unit of measurement
☐	☒	A statement on whether measurements were taken from distinct samples or whether the same sample was measured repeatedly
☐	☒	The statistical test(s) used AND whether they are one- or two-sided<br>*Only common tests should be described solely by name; describe more complex techniques in the Methods section.*
☐	☒	A description of all covariates tested
☐	☒	A description of any assumptions or corrections, such as tests of normality and adjustment for multiple comparisons
☐	☒	A full description of the statistical parameters including central tendency (e.g. means) or other basic estimates (e.g. regression coefficient) AND variation (e.g. standard deviation) or associated estimates of uncertainty (e.g. confidence intervals)
☐	☒	For null hypothesis testing, the test statistic (e.g. *F*, *t*, *r*) with confidence intervals, effect sizes, degrees of freedom and *P* value noted<br>*Give P values as exact values whenever suitable.*
☒	☐	For Bayesian analysis, information on the choice of priors and Markov chain Monte Carlo settings
☒	☐	For hierarchical and complex designs, identification of the appropriate level for tests and full reporting of outcomes
☒	☐	Estimates of effect sizes (e.g. Cohen's *d*, Pearson's *r*), indicating how they were calculated

Our web collection on statistics for biologists contains articles on many of the points above.

Software and code

Policy information about availability of computer code

Data collection	AMBER20 (for classical simulations).
Data analysis	AMBER20 (for classical simulations). WHAM 2.0.11 code for free energy calculations. Dijkstra algorithm. MEPSA code for minimum energy pathway analysis.

For manuscripts utilizing custom algorithms or software that are central to the research but not yet described in published literature, software must be made available to editors and reviewers. We strongly encourage code deposition in a community repository (e.g. GitHub). See the Nature Portfolio guidelines for submitting code & software for further information.

Data

Policy information about availability of data

All manuscripts must include a data availability statement. This statement should provide the following information, where applicable:

- Accession codes, unique identifiers, or web links for publicly available datasets
- A description of any restrictions on data availability
- For clinical datasets or third party data, please ensure that the statement adheres to our policy

All data are included in the article and Supplementary Information. Molecular simulations were based on two structures of the F. novicida Cas12a obtained upon NTS cleavage: (1) the cryo-EM structure EMD-0065 (PDB: 6GTG) solved at 3.27 Å resolution and (2) the X-ray structure PDB: 5NFV, solved at 2.50 Å resolution. Sequences of oligos, synthetic genes and gene fragments are provided in Supplementary Data 1. In vitro DNA cleavage assay time points data of Cas12a wild type and its mutants is provided in Supplementary Data 2 and the corresponding uncropped gel images are in the Source Data File. Atomic coordinates of the initial and

final structure of CRISPR-Cas12a along the traversal of the target strand toward the RuvC active site are available in figshare with the identifier 10.6084/m9.figshare.24978669. More details can be provided by the corresponding author upon request.

Human research participants

Policy information about studies involving human research participants and Sex and Gender in Research.

Reporting on sex and gender	This study does not involve human participants.
Population characteristics	This study does not involve human participants.
Recruitment	This study does not involve human participants.
Ethics oversight	This study does not involve human participants.

Note that full information on the approval of the study protocol must also be provided in the manuscript.

Field-specific reporting

Please select the one below that is the best fit for your research. If you are not sure, read the appropriate sections before making your selection.

[x] Life sciences [ ] Behavioural & social sciences [ ] Ecological, evolutionary & environmental sciences

For a reference copy of the document with all sections, see nature.com/documents/nr-reporting-summary-flat.pdf

Life sciences study design

All studies must disclose on these points even when the disclosure is negative.

Sample size	The sample size of all simulations was deemed appropriate as evident from the converged results presented in Figs. 2 and 3, and Supplementary Figs. 1, 2, 4, 5, 6, 7 and 9. In molecular dynamics simulations, convergence is based on ergodicity, indicating that all the microstates are accessible over the simulation time. Convergence of structural properties from the ensemble population was assessed in comparison with the experimentally known value. For the free energy simulations, the sample size was chosen for the energy profiles reaching convergence. Supplementary Fig. 12 shows well-converged free energy simulations indicating sufficient sampling.
Data exclusions	Analysis of molecular dynamics simulations has been performed on the entire ensemble population. For the calculation of the free energy profiles, the initial part of the simulations was excluded with the rationale of considering the converged trajectories. Convergence of the energy profiles are reported in the supplementary information.
Replication	Long timescale molecular simulations were performed in duplicates with consistent results arising from both the replicates reported in the main and supplementary figures. DNA cleavage data arise from three independent experiments (n = 3), with data presented as the average and standard error of mean.
Randomization	To maintain the randomization, each replica of molecular dynamics simulations was started with random velocities. The replicates starting from the different initial velocities converged to similar results.
Blinding	Blinding couldn't be implemented in our case because the identity (the chemical characteristics) of each sample needed to be known in the major part of the simulations and experiments that we performed.

Reporting for specific materials, systems and methods

We require information from authors about some types of materials, experimental systems and methods used in many studies. Here, indicate whether each material, system or method listed is relevant to your study. If you are not sure if a list item applies to your research, read the appropriate section before selecting a response.

Materials & experimental systems

n/a	Involved in the study	
[x]	[ ]	Antibodies
[x]	[ ]	Eukaryotic cell lines
[x]	[ ]	Palaeontology and archaeology
[x]	[ ]	Animals and other organisms
[x]	[ ]	Clinical data
[x]	[ ]	Dual use research of concern

Methods

n/a	Involved in the study	
[x]	[ ]	ChIP-seq
[x]	[ ]	Flow cytometry
[x]	[ ]	MRI-based neuroimaging
