# Supplementary material for: An alpha-helical lid guides the target DNA toward catalysis in CRISPR-Cas12a
Source: Nat Commun. 2024 Feb 17;15:1473. doi: 10.1038/s41467-024-45762-6 (PMC10874386; doi:10.1038/s41467-024-45762-6)
Supplement: Supplementary file 7 — Source data [file 41467_2024_45762_MOESM7_ESM.zip › 417323_3_data_set_8548234_s7c2xb(1).docx]

**Source Data**

**An Alpha-helical Lid Guides the Target DNA toward Catalysis in CRISPR-Cas12a**

Aakash Saha, Mohd Ahsan,^†^ Pablo R. Arantes,^†^ Michael Schmitz, Christelle Chanez, Martin Jinek and Giulia Palermo*


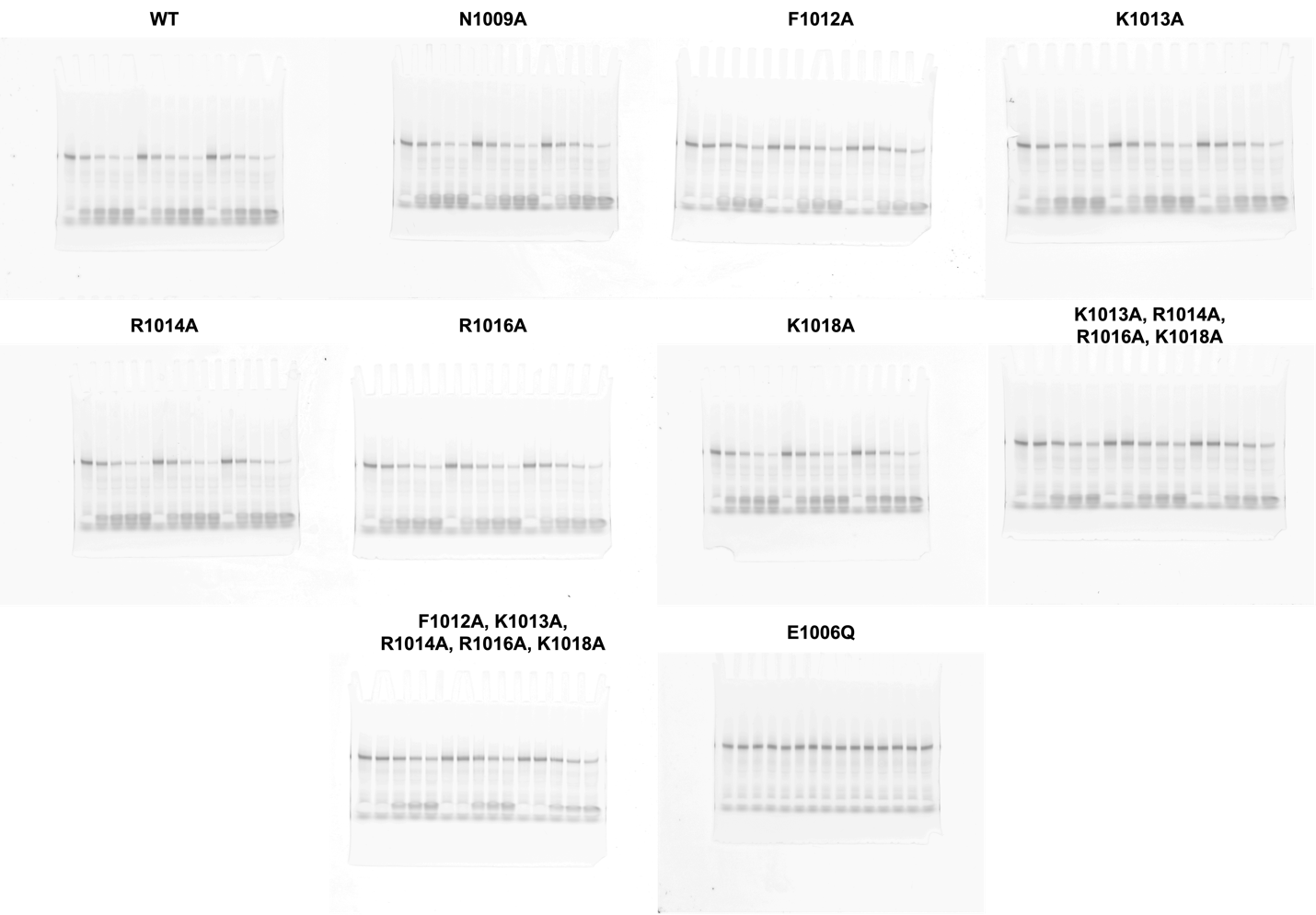


**Source Data 1: *In vitro* DNA TS cleavage assay of Cas12a mutants.** Uncropped images of denaturing polyacrylamide gels showing DNA TS cleaved products by Cas12a as used for Supplementary Fig. 17 and 18.
